# Estimated Relative Effectiveness and Public Health Impact of Cell‐Based Versus Egg‐Based Influenza Vaccines During the 2022–2023 Season in the United States

**DOI:** 10.1111/irv.70180

**Published:** 2025-11-04

**Authors:** Alicia N. Stein, Anusorn Thanataveerat, Kimberly W. McDermott, Alex Dean, Stephanie Wall, Cory Pack, Sheena G. Sullivan, Ian McGovern, Mendel D. M. Haag

**Affiliations:** ^1^ CSL Seqirus Melbourne Australia; ^2^ Veradigm Chicago Illinois USA; ^3^ School of Clinical Sciences Monash University Melbourne Australia; ^4^ CSL Seqirus Waltham Massachusetts USA; ^5^ CSL Seqirus Amsterdam The Netherlands

**Keywords:** cell‐based quadrivalent influenza vaccine, influenza, influenza burden, public health, United States, vaccine effectiveness

## Abstract

**Background:**

Egg adaptation can reduce the effectiveness of egg‐based influenza vaccines. Previous studies have demonstrated improved effectiveness of cell versus egg‐based quadrivalent influenza vaccines (QIVc and QIVe, respectively) during the 2017–2020 influenza seasons among persons aged ≥ 4 years. Here we evaluate the relative vaccine effectiveness (rVE) of QIVc versus QIVe in preventing test‐confirmed influenza among persons aged 6 months to 64 years during the US 2022–2023 influenza season, along with the potential impact on influenza burden averted.

**Methods:**

A retrospective test‐negative design was applied to linked electronic health records and claims data from QIVc or QIVe recipients who were tested for influenza in routine outpatient care within 7 days of an acute respiratory or febrile illness. rVE was estimated by comparing the odds of testing positive among QIVc versus QIVe recipients, adjusted using doubly robust methodology. The influenza burden additionally averted by vaccination with QIVc versus QIVe was estimated using a published model.

**Results:**

Of 43,086 patients included, 18.6% received QIVc and 81.4% received QIVe. The rVE of QIVc versus QIVe was 7.7% (95% CI, 0.9%–13.9%). Use of QIVc instead of QIVe during the 2022–23 influenza season would have prevented an additional 636,209 symptomatic cases of influenza, 314,130 outpatient visits, and 3759 hospitalizations.

**Conclusions:**

QIVc was superior to QIVe in the prevention of test‐confirmed influenza among persons aged 6 months to 64 years during the US 2022–2023 influenza season. The rVE of 7.7% would translate to a substantially reduced influenza burden if QIVc were used over QIVe.

## 1 Introduction

Influenza vaccination is the primary means of reducing the burden of influenza [1, 2, 3, 4]. However, the majority of the global supply of influenza vaccines is manufactured using fertilized hens' eggs. This can result in the selection of egg‐adapted viruses with mutations in the hemagglutinin (HA) protein that can alter the antigenicity of the vaccine and reduce the immune response of vaccinated persons to the circulating virus strains, thereby reducing vaccine effectiveness [5, 6, 7, 8, 9]. Use of non–egg‐based methods of vaccine production, such as propagation of candidate vaccine viruses (CVVs) in mammalian cell cultures or synthesis of recombinant HA within insect cell lines, avoids potential antigenic mismatches due to egg adaptation [5, 10].

A significant body of evidence supports the benefit of cell‐based over egg‐based influenza vaccines. This includes six retrospective cohort studies and a large multiseason retrospective test negative design (TND) study that consistently demonstrated superior effectiveness during the 2017–2018, 2018–2019, and 2019–2020 influenza seasons in the US [11, 12, 13, 14, 15, 16, 17, 18]. These findings are further supported by prospective TND studies with point estimates that generally favored cell‐ versus egg‐based vaccines during the 2017–2018 influenza season, albeit with insufficient sample sizes to estimate relative vaccine effectiveness (rVE) with high precision [19, 20, 21, 22].

The above studies included populations aged ≥ 4 years, consistent with the registered indication of the cell‐based quadrivalent influenza vaccine (QIVc; Flucelvax Quadrivalent, CSL Seqirus USA Inc., Summit, NJ, USA) at the time. In October 2021, the indication for QIVc was expanded to individuals aged ≥ 6 months in the US, providing the opportunity to evaluate the rVE in studies including the younger pediatric population. This study estimated the rVE of QIVc versus egg‐based quadrivalent influenza vaccine (QIVe) in preventing test‐confirmed influenza among individuals aged 6 months through 64 years in the outpatient care setting during the 2022–2023 influenza season in the US. Additionally, we estimated the burden of influenza that would be averted through the use of QIVc versus QIVe in this population.

## 2 Methods

### 2.1 Relative Vaccine Effectiveness

#### 2.1.1 Study Design

We estimated the rVE of QIVc versus QIVe against test‐confirmed influenza among persons aged 6 months through 64 years during the 2022–2023 influenza season in the US using a retrospective TND study, as previously described [18]. The study was designed, implemented, and reported in accordance with Good Pharmacoepidemiological Practice, applicable local regulations, and the ethical principles laid down in the Declaration of Helsinki as well as the REporting of studies Conducted using Observational Routinely‐collected health Data (RECORD).

#### 2.1.2 Data Sources

This analysis was conducted using data from the Veradigm Integrated Dataset, which includes data from US primary care electronic health record (EHR) platforms linked with Komodo Health pharmacy and medical claims data from > 60 million patients per year in the US [12, 18]. This linked, deidentified dataset is compliant with the Health Insurance Portability and Accountability Act (HIPAA) and statistically certified for research use.

#### 2.1.3 Study Population

The study population included persons aged 6 months to 64 years at vaccination because, in the US, adjuvanted and higher dose vaccines are preferentially recommended for older adults, restricting use of the cell‐based vaccine in the population 65 years and older. Eligible subjects were required to have a valid influenza test (Table S1) within 7 days of a diagnosis of an acute respiratory or febrile illness (ARFI) (Table S2) and ≥ 14 days after vaccination with either QIVc or QIVe (Table S3). Noting the CDC recommendation that children aged 6 months through 8 years who have never received a seasonal influenza vaccine or for whom vaccination history is unknown should receive two doses of influenza vaccines at least 4 weeks apart [23], we required children aged < 9 years to have a record of a previous influenza vaccination in an earlier influenza season or to have received two homologous doses of either QIVc or QIVe, given ≥ 28 days apart, during the 2022–2023 influenza season. Vaccines had to be administered between August 1, 2022, and May 6, 2023. Subjects older than 1 year were required to have EHR transcript and claims activity in the period extending back ≥ 12 months before the date of vaccination, while those aged 6 months to 1 year were required to have ≥ 6 months EHR transcript and claims activity history.

Subjects were excluded if they tested positive for influenza between May 22, 2022, and October 2, 2022; if they received more than the indicated number of influenza vaccine doses between August 1, 2022, and the date of their influenza test; or if they had received any type of influenza vaccine between May 22, 2022, and July 31, 2022. Subjects with missing sex or geographic data in both the EHR and Komodo claims databases were also excluded.

#### 2.1.4 Outcome Definitions

Influenza test results were classified as “Negative /Not Detected” or “Positive/Detected” (Supplementary Materials, Influenza Test Data Mapping). A case was defined by a positive influenza test result and a control by a negative test and no positive tests within the season timeframe (Supplementary Materials, Outcome Definitions). Molecular (e.g., polymerase chain reaction [PCR]) and antigen influenza tests were identified using current procedural terminology (CPT) or Logical Observation Identifiers Names and Codes (LOINC) codes (Table S1). Antibody and culture tests were excluded to avoid potential bias due to the lack of specificity for antibody tests to reliably detect acute disease and potential differences in patients tested by culture methods.

#### 2.1.5 Statistical Analyses

A logistic regression model was used to obtain the odds ratio (OR) comparing the odds of testing positive for influenza among QIVc recipients with the odds among QIVe recipients. rVE was calculated using the formula rVE = (1 − OR) × 100% and reported with 95% confidence intervals (CIs). The analysis was adjusted using a doubly robust approach combining inverse probability of treatment weighting (IPTW) and multivariable adjustment as described previously [18]. Unadjusted rVE was also estimated. For a complete description of statistical methods, see Supplementary Materials, Statistical Analyses, and Table S4.

The propensity score models used to derive the IPTW weights were adjusted for the following five a priori defined covariates: age (as spline), sex, geographic region as defined by the US Department of Health and Human Services (HHS) (Table S5), influenza test date (as spline), and receipt of COVID‐19 vaccine within 6 months prior to the test date. The latter was included to reduce potential confounding due to correlation between influenza and COVID‐19 vaccination decisions [24]. Analyses were also adjusted by any other covariates shown to be imbalanced between vaccine exposure groups before weighting. Other covariates assessed included race, ethnicity, week of vaccination, influenza test type, insurance/payer, Charlson Comorbidity Index (CCI) score [25], the presence of high‐risk medical conditions for which the US Centers for Disease Control and Prevention (CDC) recommends influenza vaccination, and healthcare resource utilization (Table S4). Covariate balance between vaccine exposure groups was evaluated among the controls using standardized mean differences (SMD), with an a priori set threshold of an absolute value |≤0.1| indicating a conservative negligible difference. Propensity scores for treatment group membership (i.e., QIVc versus QIVe) were first estimated among controls and the fitted model was used to calculate propensity for treatment group membership scores for the cases. Propensity scores were used to calculate stabilized weights. For the doubly robust analysis, the IPTW sample was used in a multivariable logistic regression model adjusted for the five a priori defined covariates and any other covariates that remained imbalanced after weighting (Table S4).

Three sensitivity analyses were performed. The “propensity to be tested” analysis addressed the potential bias of which patients are given an influenza test through additional adjustment by the inverse of the “propensity to be tested” score. The “matched on test week” analysis examined if there was residual confounding associated with the timing of the test, matching each case with up to five controls who were tested for influenza during the same week of the calendar year. This approach is less sensitive to model misspecification and ensured controls were selected only for weeks during which cases were present. The “seasonal peak period” analysis maximized the positive predictive value of the influenza tests by restricting the outcome measurement to the November 6 to December 24, 2022, peak period, defined using the moving epidemic method [26].

### 2.2 Burden Averted Model

The additional burden of influenza that would be averted if QIVc were used in lieu of QIVe to vaccinate persons aged 0–64 years against influenza was estimated according to previously described methodology [27]. Briefly, the burden averted modeling method used by the CDC was adapted for use in an rVE context and assessed two scenarios: one in which all vaccinated individuals received QIVe and another where all vaccinated individuals received QIVc. The additional burden averted was calculated as the number of outcomes prevented in the QIVc scenario minus the number of outcomes prevented in the QIVe scenario. The model used CDC data on influenza vaccine uptake, influenza incidence, and influenza‐related healthcare resource utilization and deaths from the 2022–2023 influenza season (Table S6). CDC estimates of absolute vaccine effectiveness (aVE; i.e., effectiveness relative to no vaccination) from the Virtual SARS‐CoV‐2, Influenza, and Other respiratory viruses Network (VISION) Outpatient network for any vaccine type were used as a proxy for the aVE of QIVe, given that QIVe represents the majority of vaccines used in this age group in the US (Table S6) [28]. The aVE of QIVc was estimated as described by Lewis et al. [29] by applying the rVE estimated in the primary analysis to the aVE of QIVe. The base case model applied the estimates, with deterministic and probabilistic sensitivity analyses exploring the impact of uncertainty.

## 3 Results

### 3.1 Study Population

**FIGURE 1:**
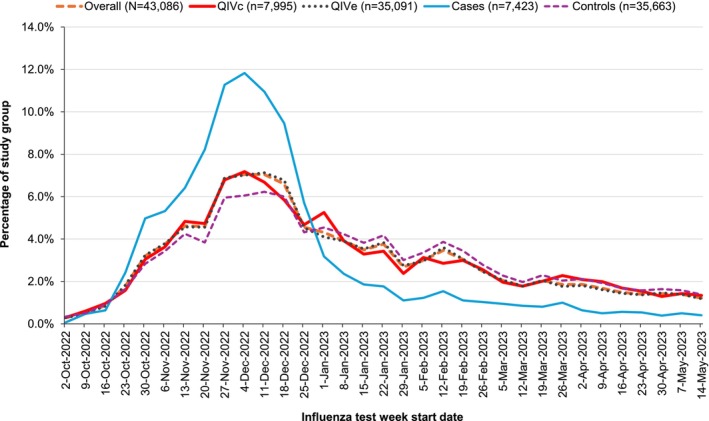
Influenza test week in the overall study population, in the QIVc and QIVe groups (independent of test result), and in cases and controls (independent of vaccine group). The *y* axis represents the percentage from each group shown in the legend. QIVc, cell‐based quadrivalent influenza vaccine; QIVe, egg‐based quadrivalent influenza vaccine.

Out of a total of 19,145,882 individuals vaccinated with either QIVc or QIVe, 43,086 met study selection criteria (Figure S1), including 1033 (13%) cases and 6962 (87%) controls in the QIVc group, and 6390 (18%) cases and 28,701 (82%) controls in the QIVe group. Timing of influenza testing was similar for both vaccine groups across the season, peaking in mid‐December 2022, with cases representing a higher proportion of tests during the seasonal peak period (Figure 1).

**FIGURE 2:**
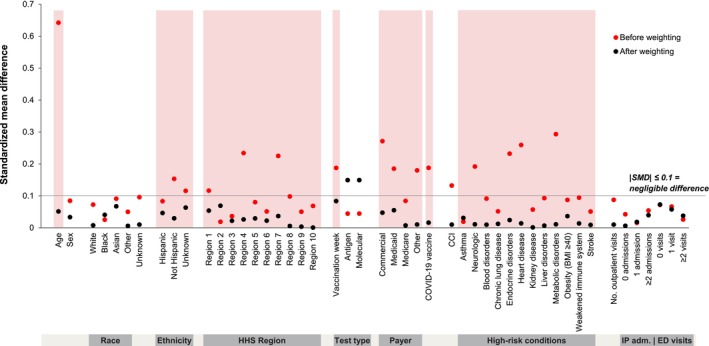
Covariate balance between vaccine groups among test negative controls, before and after weighting. “Neurologic” includes neurologic and developmental conditions; “Heart disease” includes heart disease and related conditions. BMI, body mass index, in kg/m^2^; CCI, Charlson comorbidity index; ED, emergency department; HHS, US Department of Health and Human Services; IP, inpatient; SMD, standardized mean difference.

The demographic and clinical characteristics of the full study population included in the primary analysis and in the propensity to be tested sensitivity analysis are presented in Table S7. Prior to weighting, QIVc recipients tended to be older and have higher CCI scores than QIVe recipients, and QIVc recipients were also more likely than QIVe recipients to be non‐Hispanic, to reside in HHS Region 4, to have private insurance, and to have received a COVID‐19 vaccine within the previous 6 months. The QIVe group included a higher proportion of patients residing in HHS Regions 1 and 7; QIVe recipients were also more likely to have unknown ethnicity and have government‐paid coverage (Table S7). After weighting, the only imbalance between the groups was influenza test type (SMD = 0.15) (Figure 2).

### 3.2 Relative Vaccine Effectiveness

**FIGURE 3:**
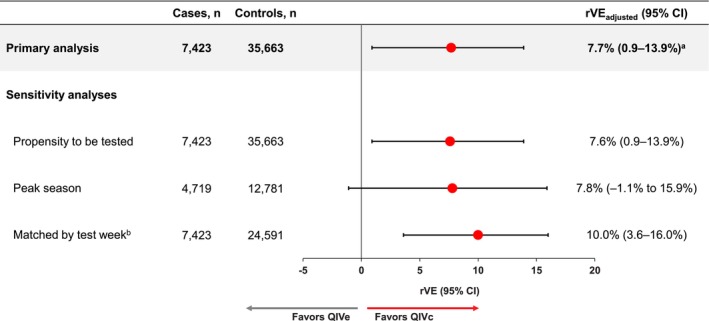
rVE of QIVc versus QIVe in prevention of test‐confirmed influenza adjusted using a doubly robust approach combining IPTW and multivariable adjustment. rVE models were adjusted for age (as spline), sex, geographic region (Department of Health and Human Services), influenza test date (as spline), and COVID‐19 vaccination as well as other imbalanced covariates (Table S4). ^a^Unadjusted rVE: 33.4% (95% CI, 28.5%–37.9%). ^b^Cases were matched to up to 5 controls, including 22% matched 1:2, 48% matched 1:3, 7% matched 1:4, and 23% matched 1:5. CI, confidence interval; IPTW, inverse probability of treatment weighting; QIVc, cell‐based quadrivalent influenza vaccine; QIVe, egg‐based quadrivalent influenza vaccine; rVE, relative vaccine effectiveness.

The adjusted rVEs obtained in the primary as well as the sensitivity analyses are presented in Figure 3. In the primary analysis, the adjusted rVE against any influenza was 7.7% (95% CI, 0.9%–13.9%).

The results of the propensity to be tested sensitivity analysis were similar, with an adjusted rVE of 7.6% (0.9%–13.9%) (Figure 3).

The matched‐by‐test week sensitivity analysis included all the cases and 69% of the controls. In addition to the covariate imbalances observed in the primary analysis population, imbalances were observed before weighting in unknown race, residence in Region 8, and liver disorders (Figure S2). In this matched analysis, the adjusted rVE was 10.0% (3.6%–16.0%).

The seasonal peak period sensitivity analysis included 17,500 subjects who had an influenza test between November 6 and December 24, 2022, including 64% of cases and 36% of controls. The demographic and clinical characteristics were similar to those of the full population, with minor differences in covariate imbalances (Table S8, Figure S3). In this restricted population, the adjusted rVE was 7.8% (−1.1 to 15.9%).

### 3.3 Burden Averted

Using the adjusted rVE of 7.7% (0.9%–13.9%) and the base case aVE for QIVe, the estimated aVEs for QIVc for the pediatric and adult populations were 52.0% (48.5%–55.2%) and 49.2% (45.5%–52.6%), respectively. Using the base case burden averted model inputs in Table S6, QIVc was estimated to prevent 636,209 more symptomatic cases compared to QIVe among individuals 6 months to 64 years of age, with proportionate reductions in healthcare resource usage in the total population (Table 1). Children and adolescents accounted for 55% of symptomatic cases and 64% of outpatient visits averted, while adults aged 50–64 years accounted for 40% of hospitalizations and 79% of deaths averted (Table S9). Sensitivity analyses showed that the results of the burden averted model were most strongly influenced by input assumptions around rVE and around influenza‐related epidemiology and healthcare resource use in the unvaccinated population (Figures S4 and S5).

**TABLE 1:** Estimated influenza burden averted by use of QIVc versus QIVe in subjects aged 0–64 years in the US 2022–2023 influenza season.

Outcome	Outcomes prevented		Incremental outcomes prevented by QIVc
	QIVc	QIVe	
Symptomatic cases	6,249,866	5,613,657	636,209
Outpatient visits	3,076,519	2,762,389	314,130
Hospitalizations^a^	36,540	32,781	3759
ICU admissions	5554	4983	571
Deaths	1238	1109	129

Abbreviations: ICU, intensive care unit; QIVc, cell‐based quadrivalent influenza vaccine; QIVe, egg‐based cell‐based quadrivalent influenza vaccine.

^a^ Hospitalizations include both ICU and non‐ICU hospital stays.

## 4 Discussion

In this real world data study, QIVc was superior to QIVe in the prevention of test‐confirmed influenza, with an rVE of 7.7% (0.9%–13.9%) among persons aged 6 months to 64 years during the 2022–2023 influenza season in the US. Sensitivity analyses conducted to determine the rVE based on subjects' likelihood of receiving an influenza test, temporal matching of the case and control populations, and the peak period of influenza activity supported the main analysis, with rVE point estimates ranging from 7.6% to 10.0%. In a burden averted modeling analysis, use of QIVc instead of QIVe during the 2022–2023 influenza season in the US would have additionally prevented 636,209 symptomatic cases of influenza and 129 influenza deaths, substantially reducing healthcare resource utilization by 314,130 outpatient visits and 3759 hospitalizations.

The 2022–2023 influenza season was the first post‐COVID‐19 season in which influenza circulated at levels comparable to seasons prior to the pandemic. It was an early season, beginning in October 2022 and peaking in December 2022, with a 6‐week seasonal peak period [30], substantially shorter than the 3–4 month seasonal peak period observed in the 2017–2018 to 2019–2020 seasons [31]. A(H3N2) viruses predominated, accounting for approximately two‐thirds of influenza cases. A(H1N1) accounted for most other cases, with low B/Victoria activity late in the season. The 2022–2023 vaccine viruses were described as being well matched to the predominant circulating strains [32], although the A(H1N1) vaccine strain was updated for the NH 2023–2024 season, addressing drift to viruses belonging to the 6B.1A.5a.2a.1 HA clade in the postpeak period [33]. Detailed sequence analyses showed amino acid substitutions in antigenic sites and/or the receptor binding site of the HA proteins of egg‐based CVVs for all strains compared with cell isolates or the original clinical samples (Table S10) [34]. In contrast, no amino‐acid changes were detected in the corresponding cell‐based CVVs (Table S10) [35], improving the likelihood of generating immune responses better targeted to the circulating viruses.

This study adds a fourth season to the body of evidence supporting improved effectiveness of cell‐based compared to egg‐based influenza vaccines [11, 12, 13, 14, 15, 16, 17, 18] and provides the first evidence available in the extended population from 6 months to 64 years of age. Our finding of a significant benefit of cell‐based vaccine was not replicated in a cohort study conducted during the same season that reported comparable effectiveness between QIVc and QIVe in preventing hospitalized influenza in adults aged 18–49 or 50–64 years [36]. With just 112 cases, that study was underpowered to detect a significant rVE of plausible magnitude and reported divergent estimates with very wide CIs for the two adult age groups studied, complicating interpretation. Conversely, our study used a large integrated dataset in which 7423 cases were identified, permitting more precise estimation.

The rVE of 7.7% (0.9%–13.9%) is generally consistent with prior observational studies that reported the benefit of QIVc versus QIVe over the 2017–2018 to 2019–2020 influenza seasons [11, 12, 13, 14, 15, 16, 17, 18], including a large retrospective TND study using similar methodology that showed superior effectiveness of QIVc over QIVe in the population aged 4–64 years, with estimated rVEs of 14.8% (7.0%–22.0%), 12.5% (4.7%–19.6%), and 10.0% (2.7%–16.7%) in the three influenza seasons of the study, respectively [18]. The magnitudes of the estimated rVEs in each season likely reflect the seasonal characteristics of the circulating and vaccine viruses and the degree to which antigenic drift and any egg adaptations present in the vaccine virus alter their antigenic similarity. For A(H3N2), the predominant circulating strain in 2022–2023, all recommended egg‐based CVVs included the D186N and D225G egg‐adapted mutations, located in antigenic site B and the receptor binding site of the HA protein, respectively, with individual CVVs additionally including the S46P substitution in antigenic site C or the S96C substitution in antigenic site D [34]. Similarly, all recommended egg‐based CVVs for A(H1N1) exhibited the Q223R substitution in the receptor binding site, with an individual CVV additionally including the A195E substitution in antigenic site Sb, although their potential impact may have been masked by the drift documented in this vaccine strain towards the end of the season.

Results of the sensitivity analyses supported the primary analysis findings, with similar estimated rVEs and overlapping CIs. Adjusting for propensity to be tested had no impact on the estimated rVE, indicating it was not a source of bias. The analysis matching cases and controls on test week estimated a numerically higher rVE with slightly improved precision, suggesting any residual confounding due to timing of the test in the primary analysis would favor the egg‐based vaccine. The seasonal peak period rVE estimate was consistent with the primary analysis, suggesting an appropriate positive predictive value of the tests throughout the season. However, because of the early and narrow peak observed in the 2022–2023 influenza season, the included population dropped by 60%, resulting in a wider CI that spanned the null.

The public health impact of improved vaccines such as QIVc depends on both the absolute effectiveness of the standard vaccine and the severity of the season [29, 37]. The 2022–2023 influenza season was classified as having moderate severity, with high severity among children [38]. Our analyses show that even with a seemingly modest rVE of 7.7%, use of QIVc instead of QIVe for all vaccinated individuals aged < 65 years would translate into a substantial public health benefit, preventing 636,209 additional symptomatic cases of influenza and 129 influenza deaths, as well as reducing related health care resource use. The burden averted is likely underestimated, since the model conservatively applies the CDC influenza burden estimates that correspond to the general population (including both vaccinated and unvaccinated individuals) to the unvaccinated population and does not consider the potential indirect effect of vaccination through decreased transmission [27].

## 5 Strengths and Limitations

The use of test‐confirmed influenza outcomes in temporal proximity to a diagnosis of acute respiratory or febrile illness is a strength, important for ruling out COVID‐19 and other co‐circulating respiratory viruses and ensuring similar healthcare‐seeking behavior for cases and controls. However, influenza tests were performed as part of routine outpatient care rather than according to prespecified criteria, and most were rapid antigen tests, which can have lower test sensitivity and specificity than PCR [39]. Nevertheless, misclassification of cases as controls, if nondifferential, should only minimally harm vaccine effectiveness estimates [40], and the Food and Drug Administration (FDA) requirement that rapid tests have at least 95% specificity minimizes the risk of misclassification of controls as cases [41]. Furthermore, available data for the influenza tests did not allow discrimination by viral type or subtypes, with all results being classified as positive or negative for any influenza. Another limitation was that vaccination was not randomly assigned; thus, unmeasured and residual confounding remain potential sources of bias, as in all observational research. The study methodology leveraged all available data to adjust for measured confounders, but clinical and claims data sources can be incomplete (e.g., race and ethnicity) and do not include individual or contextual socioeconomic data that could inform health‐seeking behavior [42]. In addition, because age‐specific rVE could not be generated in the study, the burden averted model applied the same rVE of QIVc versus QIVe across all age groups (Table S6). Finally, we did not evaluate the rVE of QIVc against other vaccines used in the population under 65 years, such as the live attenuated influenza vaccine (LAIV) or the recombinant influenza vaccine (RIV) because their uptake in 2022–2023 was too low to enable robust, appropriately powered comparative analyses. These vaccines also have more restricted indications, further impacting feasibility.

## 6 Conclusions

In this large‐scale, retrospective TND analysis of the 2022–2023 influenza season, QIVc demonstrated superior effectiveness in preventing test‐confirmed influenza compared to QIVe among individuals 6 months to 64 years of age in the US, with an estimated rVE of 7.7%. Based on this rVE, use of QIVc instead of QIVe would have prevented 636,209 additional symptomatic cases of influenza and an associated 314,130 outpatient visits, 3759 hospitalizations, and 129 deaths in this population.

## Author Contributions

**Alicia N. Stein:** conceptualization, methodology, formal analysis, funding acquisition, writing – original draft, writing – review and editing, investigation. **Anusorn Thanataveerat:** conceptualization, methodology, formal analysis, writing – review and editing, investigation. **Kimberly W. McDermott:** conceptualization, methodology, formal analysis, writing – review and editing, investigation. **Alex Dean:** data curation, formal analysis, writing – original draft. **Stephanie Wall:** conceptualization, methodology, formal analysis, writing – review and editing, investigation. **Cory Pack:** data curation, formal analysis, writing – review and editing. **Sheena G. Sullivan:** conceptualization, methodology, formal analysis, writing – review and editing. **Ian McGovern:** formal analysis, writing – review and editing. **Mendel D. M. Haag:** conceptualization, methodology, formal analysis, writing – review and editing.

## Ethics Statement

The study was conducted using a database linking deidentified electronic medical records with medical and pharmacy claims data. The database operates under a certification of statistical deidentification by a third party HIPAA Expert Determination provider. The certification process ensures that users only have access to deidentified data and cannot pose a risk to reidentification. Because the database is HIPAA‐certified based on validated independent assessment and users receive unidentifiable/deidentified or coded data (with identifiers kept separately), the study does not fall within the regulatory definition of research involving human participants as outlined by the Code of Federal Regulations (policy 46.102(f)). The study is therefore exempt from institutional review board approval, and informed consent was not needed. The study was designed, implemented, and reported in accordance with the ISPE Guidelines for Good Pharmacoepidemiological Practice, with applicable local regulations, with the ethical principles laid down in the Declaration of Helsinki of 1964 and its later amendments, and with the principles of REporting of studies Conducted using Observational Routinely collected health Data (RECORD).

## Conflicts of Interest

AS, IM, and MH are employees of CSL Seqirus. AT, KM, ad, SW, and CP are employees of Veradigm. SGS reports consulting for CSL Behring, Moderna, Pfizer, Novavax, Evo Health, Astra Zeneca, GSK, and Sanofi.

## Supporting information


**Table S1:** Influenza test codes.
**Table S2:** ICD‐10‐CM used to identify acute respiratory or febrile illnesses.
**Table S3:** CVX, CPT, and NDC Codes for 2022–2023 influenza vaccines.
**Table S4:** Summary of statistical analyses.
**Table S5:** HHS regions.
**Table S6:** Model input data for analysis of influenza burden averted.
**Figure S1:** Subject selection.
**Table S7:** Demographic and clinical characteristics of the overall study population.
**Figure S2:** Covariate balance of controls in the matched on week population before and after weighting.
**Table S8:** Demographic and clinical characteristics of the peak season study population.
**Figure S3:** Covariate balance of controls in the peak period population before and after weighting.
**Table S9:** Additional outcomes prevented by use of QIVc versus QIVe, by age group.
**Figure S4:** Deterministic sensitivity analysis of uncertainty around the base‐case results of the analysis of symptomatic cases averted.
**Figure S5:** Probabilistic sensitivity analysis.
**Table S10:** Amino acid substitutions in the HA protein of cell‐based and egg‐based vaccine viruses, NH 2022–2023 season.

## Data Availability

The datasets generated and/or analyzed during the current studies were used under license from Veradigm and are not publicly available. Upon request and subject to review, CSL Seqirus may provide the deidentified aggregate‐level data that support the findings of this study. Deidentified data (including participant data as applicable) may be shared upon approval of an analysis proposal and a signed data access agreement.
