# Supplementary material for: Estimated Relative Effectiveness and Public Health Impact of Cell‐Based Versus Egg‐Based Influenza Vaccines During the 2022–2023 Season in the United States
Source: Influenza Other Respir Viruses. 2025 Nov 4;19(11):e70180. doi: 10.1111/irv.70180 (PMC12583930; doi:10.1111/irv.70180)
Supplement: Supplementary file 1 — Table S1: Influenza test codes. Table S2: ICD‐10‐CM used to identify acute respiratory or febrile illnesses. Table S3: CVX, CPT, and NDC Codes for 2022–2023 influenza vaccines. Table S4: Summary of statistical analyses. Table S5: HHS regions. Table S6: Model input data for analysis of influenza burden averted. Figure S1: Subject selection. Table S7: Demographic and clinical characteristics of the overall study population. Figure S2: Covariate balance of controls in the matched on week population before and after weighting. Table S8: Demographic and clinical characteristics of the peak season study population. Figure S3: Covariate balance of controls in the peak period population before and after weighting. Table S9: Additional outcomes prevented by use of QIVc versus QIVe, by age group. Figure S4: Deterministic sensitivity analysis of uncertainty around the base‐case results of the analysis of symptomatic cases averted. Figure S5: Probabilistic sensitivity analysis. Table S10: Amino acid substitutions in the HA protein of cell‐based and egg‐based vaccine viruses, NH 2022–2023 season. [file IRV-19-e70180-s001.docx]

**Supplementary Materials for**

**Estimated relative effectiveness and public health impact of cell-based versus egg-based influenza vaccines during the 2022–2023 season in the United States**

Alicia N. Stein, Anusorn Thanataveerat, Kimberly W. McDermott, Alex Dean, Stephanie Wall, Cory Pack, Sheena G. Sullivan, Ian McGovern, Mendel D. M. Haag

Table of Contents

[Table S1. Influenza test codes 2](#_Toc209523527)

[Table S2. ICD-10-CM used to identify acute respiratory or febrile illnesses 7](#_Toc209523528)

[Table S3. CVX, CPT, and NDC Codes for 2022–2023 influenza vaccines 14](#_Toc209523529)

[Influenza Test Data Mapping 15](#_Toc209523530)

[Outcome Definitions 17](#_Toc209523531)

[Statistical Analysis 19](#_Toc209523532)

[Table S4. Summary of statistical analyses 25](#_Toc209523533)

[Table S5. HHS regions. 28](#_Toc209523534)

[Table S6. Model input data for analysis of influenza burden averted 29](#_Toc209523535)

[Figure S1. Subject selection 30](#_Toc209523536)

[Figure S2. Covariate balance of controls in the matched on week population before and after weighting 34](#_Toc209523537)

[Table S8. Demographic and clinical characteristics of the peak season study population 35](#_Toc209523538)

[Figure S3. Covariate balance of controls in the peak period population before and after weighting 38](#_Toc209523539)

[Table S9. Additional outcomes prevented by use of QIVc vs. QIVe, by age group 39](#_Toc209523540)

[Figure S4. Deterministic sensitivity analysis of uncertainty around the base-case results of the analysis of symptomatic cases averted 40](#_Toc209523541)

[Figure S5. Probabilistic sensitivity analysis 42](#_Toc209523542)

[Table S10. Amino acid substitutions in the HA protein of cell-based and egg-based vaccine viruses, NH 2022–2023 season 43](#_Toc209523543)

Table S1. Influenza test codes

| **Code type** | **Code** | **Long Name** | **Test Type** |
| --- | --- | --- | --- |
| CPT | 87275 | Infectious agent antigen detection by immunofluorescent technique; influenza B virus | Ag |
| CPT | 87276 | Infectious agent antigen detection by immunofluorescent technique; influenza A virus | Ag |
| CPT | 87400 | Infectious agent antigen detection by immunoassay technique, (e.g., EIA, ELISA, FIA, IMCA) qualitative or semiquantitative; Influenza, A or B, each | Ag |
| CPT | 87501 | Infectious agent detection by nucleic acid (DNA or RNA); influenza virus, includes reverse transcription, when performed, and amplified probe technique, each type or subtype | M |
| CPT | 87502 | Infectious agent detection by nucleic acid (DNA or RNA); influenza virus, for multiple types or sub- types, includes multiplex reverse transcription, when performed, and multiplex amplified probe technique, first 2 types or sub-types | M |
| CPT | 87503 | Infectious agent detection by nucleic acid (DNA or RNA); influenza virus, for multiple types or sub- types, includes multiplex reverse transcription, when performed, and multiplex amplified probe technique, each additional influenza virus type or sub-type beyond 2 (List separately in addition to code for primary procedure) | M |
| CPT | 87804 | Infectious agent antigen detection by immunoassay with direct optical observation; Influenza | Ag |
| LOINC | 100343-3 | Influenza virus B RNA [Presence] in Saliva (oral fluid) by NAA with probe detection | M |
| LOINC | 100344-1 | Influenza virus A RNA [Presence] in Saliva (oral fluid) by NAA with probe detection | M |
| LOINC | 22825-4 | Influenza virus A Ag [Presence] in Specimen by Immune diffusion (ID) | Ag |
| LOINC | 24015-0 | Influenza virus A+B Ag [Presence] in Specimen | Ag |
| LOINC | 31858-4 | Influenza virus A Ag [Presence] in Throat | Ag |
| LOINC | 31859-2 | Influenza virus A Ag [Presence] in Specimen | Ag |
| LOINC | 31860-0 | Influenza virus A+B Ag [Presence] in Throat | Ag |
| LOINC | 31861-8 | Influenza virus A+B+C Ag [Presence] in Throat | Ag |
| LOINC | 31862-6 | Influenza virus A+B+C Ag [Presence] in Specimen | Ag |
| LOINC | 31863-4 | Influenza virus B Ag [Presence] in Throat | Ag |
| LOINC | 31864-2 | Influenza virus B Ag [Presence] in Specimen | Ag |
| LOINC | 33535-6 | Influenza virus A+B Ag [Presence] in Nasopharynx | Ag |
| LOINC | 34487-9 | Influenza virus A RNA [Presence] in Specimen by NAA with probe detection | M |
| LOINC | 38381-0 | Influenza virus A cDNA [Presence] in Specimen by NAA with probe detection | M |
| LOINC | 40981-3 | Deprecated Influenza virus A RNA [Presence] in Unspecified specimen by Probe & target amplification method | M |
| LOINC | 40982-1 | Influenza virus B RNA [Presence] in Specimen by NAA with probe detection | M |
| LOINC | 43874-7 | Influenza virus A Ag [Presence] in Nasopharynx | Ag |
| LOINC | 43895-2 | Influenza virus B Ag [Presence] in Nasopharynx | Ag |
| LOINC | 44558-5 | Influenza virus A Ag [Presence] in Nasopharynx by Immunofluorescence | Ag |
| LOINC | 44559-3 | Influenza virus A Ag [Presence] in Bronchial specimen by Immunofluorescence | Ag |
| LOINC | 44560-1 | Influenza virus A Ag [Presence] in Nose by Immunofluorescence | Ag |
| LOINC | 44561-9 | Influenza virus A Ag [Presence] in Trachea by Immunofluorescence | Ag |
| LOINC | 44562-7 | Influenza virus A Ag [Presence] in Bronchial specimen | Ag |
| LOINC | 44563-5 | Influenza virus A Ag [Presence] in Nose | Ag |
| LOINC | 44564-3 | Influenza virus A Ag [Presence] in Nose by Immunoassay | Ag |
| LOINC | 44566-8 | Influenza virus A+B Ag [Presence] in Bronchial specimen | Ag |
| LOINC | 44567-6 | Influenza virus A+B Ag [Presence] in Nose | Ag |
| LOINC | 44571-8 | Influenza virus B Ag [Presence] in Nasopharynx by Immunofluorescence | Ag |
| LOINC | 44572-6 | Influenza virus B Ag [Presence] in Bronchial specimen by Immunofluorescence | Ag |
| LOINC | 44573-4 | Influenza virus B Ag [Presence] in Nose by Immunofluorescence | Ag |
| LOINC | 44574-2 | Influenza virus B Ag [Presence] in Trachea by Immunofluorescence | Ag |
| LOINC | 44575-9 | Influenza virus B Ag [Presence] in Nose by Immunoassay | Ag |
| LOINC | 44576-7 | Influenza virus B Ag [Presence] in Bronchial specimen | Ag |
| LOINC | 44577-5 | Influenza virus B Ag [Presence] in Nose | Ag |
| LOINC | 46082-4 | Influenza virus A Ag [Presence] in Nasopharynx by Immunoassay | Ag |
| LOINC | 46083-2 | Influenza virus B Ag [Presence] in Nasopharynx by Immunoassay | Ag |
| LOINC | 48509-4 | Influenza virus A and B RNA [Identifier] in Specimen by NAA with probe detection | M |
| LOINC | 49520-0 | Influenza virus A H1 RNA [Presence] in Isolate by NAA with probe detection | M |
| LOINC | 49521-8 | Influenza virus A H1 RNA [Presence] in Specimen by NAA with probe detection | M |
| LOINC | 49522-6 | Influenza virus A H3 Ag [Presence] in Isolate by Immunofluorescence | Ag |
| LOINC | 49523-4 | Influenza virus A H3 RNA [Presence] in Isolate by NAA with probe detection | M |
| LOINC | 49524-2 | Influenza virus A H3 RNA [Presence] in Specimen by NAA with probe detection | M |
| LOINC | 49529-1 | Influenza virus A Ag [Presence] in Isolate by Immunofluorescence | Ag |
| LOINC | 49531-7 | Influenza virus A RNA [Presence] in Isolate by NAA with probe detection | M |
| LOINC | 49534-1 | Influenza virus B Ag [Presence] in Isolate by Immunofluorescence | Ag |
| LOINC | 49535-8 | Influenza virus B RNA [Presence] in Isolate by NAA with probe detection | M |
| LOINC | 49537-4 | Influenza virus A and B RNA [Identifier] in Isolate by NAA with probe detection | M |
| LOINC | 50697-2 | Influenza virus A Ag [Identifier] in Isolate | Ag |
| LOINC | 50701-2 | Influenza virus A H1 Ag [Presence] in Isolate by Immunofluorescence | Ag |
| LOINC | 50707-9 | Influenza virus A polymerase B1 cDNA [Presence] in Isolate by Sequencing | M |
| LOINC | 53381-0 | Influenza virus A Ab [Identifier] in Serum | Ab |
| LOINC | 54240-7 | Influenza virus Ag [Presence] in Specimen | Ag |
| LOINC | 54241-5 | Influenza virus B Ag [Presence] in Isolate | Ag |
| LOINC | 54243-1 | Influenza virus RNA [Identifier] in Specimen by Probe | M |
| LOINC | 54244-9 | Influenza virus identified in Specimen | M |
| LOINC | 55465-9 | Influenza virus A H1 2009 pandemic RNA [Presence] in Specimen by NAA with probe detection | M |
| LOINC | 5860-2 | Influenza virus A Ag [Presence] in Throat by Immunoassay | Ag |
| LOINC | 5861-0 | Influenza virus A Ag [Presence] in Throat by Immunofluorescence | Ag |
| LOINC | 5862-8 | Influenza virus A Ag [Presence] in Specimen by Immunoassay | Ag |
| LOINC | 5863-6 | Influenza virus A Ag [Presence] in Specimen by Immunofluorescence | Ag |
| LOINC | 5864-4 | Influenza virus B Ag [Presence] in Throat by Immunoassay | Ag |
| LOINC | 5865-1 | Influenza virus B Ag [Presence] in Throat by Immunofluorescence | Ag |
| LOINC | 5866-9 | Influenza virus B Ag [Presence] in Specimen by Immunoassay | Ag |
| LOINC | 5867-7 | Influenza virus B Ag [Presence] in Specimen by Immunofluorescence | Ag |
| LOINC | 60538-6 | Influenza virus A H1+H3+B RNA [Presence] in Specimen by NAA with probe detection | M |
| LOINC | 61102-0 | Influenza virus A and B Ag [Identifier] in Specimen by Immunofluorescence | Ag |
| LOINC | 62462-7 | Influenza virus A+B RNA [Presence] in Specimen by NAA with probe detection | M |
| LOINC | 6435-2 | Influenza virus A+B Ag [Presence] in Throat by Immunoassay | Ag |
| LOINC | 6436-0 | Influenza virus A+B Ag [Presence] in Throat by Immunofluorescence | Ag |
| LOINC | 6437-8 | Influenza virus A+B Ag [Presence] in Specimen by Immunoassay | Ag |
| LOINC | 6438-6 | Influenza virus A+B Ag [Presence] in Specimen by Immunofluorescence | Ag |
| LOINC | 6439-4 | Influenza virus A+B+C Ag [Presence] in Throat by Immunoassay | Ag |
| LOINC | 6440-2 | Influenza virus A+B+C Ag [Presence] in Throat by Immunofluorescence | Ag |
| LOINC | 6441-0 | Influenza virus A+B+C Ag [Presence] in Specimen by Immunoassay | Ag |
| LOINC | 6442-8 | Influenza virus A+B+C Ag [Presence] in Specimen by Immunofluorescence | Ag |
| LOINC | 72356-9 | Influenza virus A and B Ag [Identifier] in Specimen by Rapid immunoassay | Ag |
| LOINC | 72365-0 | Influenza virus A and B Ag [Identifier] in Nose by Immunofluorescence | Ag |
| LOINC | 72366-8 | Influenza virus A and B Ag [Identifier] in Nose by Rapid immunoassay | Ag |
| LOINC | 72367-6 | Influenza virus A+B Ag [Presence] in Nose by Rapid immunoassay | Ag |
| LOINC | 74784-0 | Influenza virus B lineage RNA [Identifier] in Specimen by NAA with probe detection | M |
| LOINC | 74785-7 | Influenza virus B Victoria lineage RNA [Presence] in Specimen by NAA with probe detection | M |
| LOINC | 74786-5 | Influenza virus B Yamagata lineage RNA [Presence] in Specimen by NAA with probe detection | M |
| LOINC | 76077-7 | Influenza virus A RNA [Presence] in Bronchoalveolar lavage by NAA with probe detection | M |
| LOINC | 76078-5 | Influenza virus A RNA [Presence] in Nasopharynx by NAA with probe detection | M |
| LOINC | 76079-3 | Influenza virus B RNA [Presence] in Bronchoalveolar lavage by NAA with probe detection | M |
| LOINC | 76080-1 | Influenza virus B RNA [Presence] in Nasopharynx by NAA with probe detection | M |
| LOINC | 77026-3 | Influenza virus A H1 RNA [Presence] in Nasopharynx by NAA with probe detection | M |
| LOINC | 77027-1 | Influenza virus A H3 RNA [Presence] in Nasopharynx by NAA with probe detection | M |
| LOINC | 77028-9 | Influenza virus A H1 2009 pandemic RNA [Presence] in Nasopharynx by NAA with probe detection | M |
| LOINC | 77383-8 | Influenza virus A Ag [Presence] in Bronchoalveolar lavage by Immunofluorescence | Ag |
| LOINC | 77384-6 | Influenza virus B Ag [Presence] in Bronchoalveolar lavage by Immunofluorescence | Ag |
| LOINC | 80382-5 | Influenza virus A Ag [Presence] in Upper respiratory specimen by Rapid immunoassay | Ag |
| LOINC | 80383-3 | Influenza virus B Ag [Presence] in Upper respiratory specimen by Rapid immunoassay | Ag |
| LOINC | 80588-7 | Influenza virus A M gene [Presence] in Nasopharynx by NAA with probe detection | M |
| LOINC | 80589-5 | Influenza virus A H1 HA gene [Presence] in Nasopharynx by NAA with probe detection | M |
| LOINC | 80590-3 | Influenza virus A H3 HA gene [Presence] in Nasopharynx by NAA with probe detection | M |
| LOINC | 80591-1 | Influenza virus B NS gene [Presence] in Nasopharynx by NAA with probe detection | M |
| LOINC | 82166-0 | Influenza virus A RNA [Presence] in Nasopharynx by NAA with non-probe detection | M |
| LOINC | 82167-8 | Influenza virus A H1 RNA [Presence] in Nasopharynx by NAA with non-probe detection | M |
| LOINC | 82168-6 | Influenza virus A H1 2009 pandemic RNA [Presence] in Nasopharynx by NAA with non-probe detection | M |
| LOINC | 82169-4 | Influenza virus A H3 RNA [Presence] in Nasopharynx by NAA with non-probe detection | M |
| LOINC | 82170-2 | Influenza virus B RNA [Presence] in Nasopharynx by NAA with non-probe detection | M |
| LOINC | 82461-5 | Influenza virus A and B and H1 2009 pandemic RNA [Identifier] in Upper respiratory specimen by NAA with probe detection | M |
| LOINC | 85477-8 | Influenza virus A RNA [Presence] in Upper respiratory specimen by NAA with probe detection | M |
| LOINC | 85478-6 | Influenza virus B RNA [Presence] in Upper respiratory specimen by NAA with probe detection | M |
| LOINC | 85821-7 | Influenza virus B Victoria lineage Ag [Presence] in Isolate by Hemagglutination inhibition | Ag |
| LOINC | 86318-3 | Influenza virus B Yamagata lineage Ag [Presence] in Isolate by Hemagglutination inhibition | Ag |
| LOINC | 86565-9 | Influenza virus A Ag [Presence] in Tissue by Immunofluorescence | Ag |
| LOINC | 86568-3 | Influenza virus A RNA [Presence] in Cerebral spinal fluid by NAA with probe detection | M |
| LOINC | 86569-1 | Influenza virus A RNA [Presence] in Tissue by NAA with probe detection | M |
| LOINC | 86571-7 | Influenza virus B RNA [Presence] in Cerebral spinal fluid by NAA with probe detection | M |
| LOINC | 86572-5 | Influenza virus B RNA [Presence] in Tissue by NAA with probe detection | M |
| LOINC | 88193-8 | Influenza virus A RNA [Presence] in Cornea or Conjunctiva by NAA with probe detection | M |
| LOINC | 88194-6 | Influenza virus B Ag [Presence] in Tissue by Immunofluorescence | Ag |
| LOINC | 88195-3 | Influenza virus B RNA [Presence] in Cornea or Conjunctiva by NAA with probe detection | M |
| LOINC | 88592-1 | Influenza virus B RNA [Presence] in Lower respiratory specimen by NAA with probe detection | M |
| LOINC | 88596-2 | Influenza virus B RNA [Presence] in Pericardial fluid by NAA with probe detection | M |
| LOINC | 88599-6 | Influenza virus A RNA [Presence] in Lower respiratory specimen by NAA with probe detection | M |
| LOINC | 88600-2 | Influenza virus A RNA [Presence] in Pericardial fluid by NAA with probe detection | M |
| LOINC | 88904-8 | Influenza virus A Ag [Presence] in Lower respiratory specimen by Immunofluorescence | Ag |
| LOINC | 88905-5 | Influenza virus B Ag [Presence] in Lower respiratory specimen by Immunofluorescence | Ag |
| LOINC | 92141-1 | Influenza virus B RNA [Presence] in Respiratory specimen by NAA with probe detection | M |
| LOINC | 92142-9 | Influenza virus A RNA [Presence] in Respiratory specimen by NAA with probe detection | M |
| LOINC | 92808-5 | Influenza virus A H3 RNA [Presence] in Upper respiratory specimen by NAA with probe detection | M |
| LOINC | 92809-3 | Influenza virus A H1 RNA [Presence] in Upper respiratory specimen by NAA with probe detection | M |
| LOINC | 92976-0 | Influenza virus B RNA [Presence] in Lower respiratory specimen by NAA with non-probe detection | M |
| LOINC | 92977-8 | Influenza virus A RNA [Presence] in Lower respiratory specimen by NAA with non-probe detection | M |
| LOINC | 94394-4 | Influenza virus A H3 RNA [Presence] in Lower respiratory specimen by NAA with probe detection | M |
| LOINC | 94395-1 | Influenza virus A H1 2009 pandemic RNA [Presence] in Lower respiratory specimen by NAA with probe detection | M |
| LOINC | 94396-9 | Influenza virus A H1 RNA [Presence] in Lower respiratory specimen by NAA with probe detection | M |
| LOINC | 99623-1 | Influenza virus A N1 RNA [Presence] in Specimen by NAA with probe detection | M |

Ag, antigen; CPT, Current Procedural Terminology; EIA, enzyme immunoassay; ELISA, enzyme-linked immunosorbent assay; FIA, fluorescence immunoassay; IMCA, immunochemiluminometric assay; LOINC, Logical Observation Identifiers Names and Codes; M, molecular detection based test, including polymerase chain reaction (PCR); NAA, nucleic acid amplification.

Table S2. ICD-10-CM used to identify acute respiratory or febrile illnesses

| **Acute Respiratory or Febrile Illness^a,b^** | **ICD-10-CM Code** |
| --- | --- |
| Sepsis, unspecified organism | A41.9 |
| Viral infection of unspecified site | B34 |
| Adenovirus infection, unspecified | B34.0 |
| Enterovirus infection, unspecified | B34.1 |
| Coronavirus infection, unspecified | B34.2 |
| Parvovirus infection, unspecified | B34.3 |
| Papovavirus infection, unspecified | B34.4 |
| Other viral infection, unspecified | B34.8 |
| Viral infection, unspecified | B34.9 |
| Respiratory syncytial virus as the cause of diseases classified elsewhere | B97.4 |
| Other viral agents as the cause of diseases classified elsewhere | B97.8 |
| Human metapneumovirus as the cause of diseases classified elsewhere | B97.81 |
| Other viral agents as the cause of diseases classified elsewhere | B97.89 |
| Acute nasopharyngitis | J00 |
| Acute sinusitis | J01 |
| Acute maxillary sinusitis | J01.0 |
| Acute maxillary sinusitis, unspecified | J01.00 |
| Acute recurrent maxillary sinusitis | J01.01 |
| Acute frontal sinusitis | J01.1 |
| Acute frontal sinusitis, unspecified | J01.10 |
| Acute recurrent frontal sinusitis | J01.11 |
| Acute ethmoidal sinusitis | J01.2 |
| Acute ethmoidal sinusitis, unspecified | J01.20 |
| Acute recurrent ethmoidal sinusitis | J01.21 |
| Acute sphenoidal sinusitis | J01.3 |
| Acute sphenoidal sinusitis, unspecified | J01.30 |
| Acute recurrent sphenoidal sinusitis | J01.31 |
| Acute pansinusitis | J01.4 |
| Acute pansinusitis, unspecified | J01.40 |
| Acute recurrent pansinusitis | J01.41 |
| Other acute sinusitis | J01.8 |
| Other acute sinusitis | J01.80 |
| Other acute recurrent sinusitis | J01.81 |
| Other acute sinusitis | J01.9 |
| Acute sinusitis, unspecified | J01.90 |
| Acute recurrent sinusitis, unspecified | J01.91 |
| Acute pharyngitis | J02 |
| Streptococcal pharyngitis | J02.0 |
| Acute pharyngitis due to other specified organisms | J02.8 |
| Acute pharyngitis, unspecified | J02.9 |
| Acute tonsillitis | J03 |
| Streptococcal tonsillitis | J03.0 |
| Acute streptococcal tonsillitis, unspecified | J03.00 |
| Acute recurrent streptococcal tonsillitis | J03.01 |
| Acute tonsillitis due to other specified organisms | J03.8 |
| Acute tonsillitis due to other specified organisms | J03.80 |
| Acute recurrent tonsillitis due to other specified organisms | J03.81 |
| Acute tonsillitis, unspecified | J03.9 |
| Acute tonsillitis, unspecified | J03.90 |
| Acute recurrent tonsillitis, unspecified | J03.91 |
| Acute laryngitis and tracheitis | J04 |
| Acute laryngitis | J04.0 |
| Acute tracheitis | J04.1 |
| Acute tracheitis without obstruction | J04.10 |
| Acute tracheitis with obstruction | J04.11 |
| Acute laryngotracheitis | J04.2 |
| Supraglottitis, unspecified | J04.3 |
| Supraglottitis, unspecified, without obstruction | J04.30 |
| Supraglottitis, unspecified, with obstruction | J04.31 |
| Acute upper respiratory infections of multiple and unspecified sites | J06 |
| Acute laryngopharyngitis | J06.0 |
| Other acute upper respiratory infections of multiple sites | J06.8 |
| Acute upper respiratory infection, unspecified | J06.9 |
| Influenza due to certain identified influenza viruses | J09 |
| Influenza due to identified novel influenza A virus | J09.X |
| Influenza due to identified novel influenza A virus with pneumonia | J09.X1 |
| Influenza due to identified novel influenza A virus with other respiratory manifestations | J09.X2 |
| Influenza due to identified novel influenza A virus with gastrointestinal manifestations | J09.X3 |
| Influenza due to identified novel influenza A virus with other manifestations | J09.X9 |
| Influenza due to other identified influenza virus | J10 |
| Influenza due to other identified influenza virus with pneumonia | J10.0 |
| Influenza due to other identified influenza virus with unspecified type of pneumonia | J10.00 |
| Influenza due to other identified influenza virus with the same other identified influenza virus pneumonia | J10.01 |
| Influenza due to other identified influenza virus with other specified pneumonia | J10.08 |
| Influenza due to other identified influenza virus with other respiratory manifestations | J10.1 |
| Influenza due to other identified influenza virus with gastrointestinal manifestations | J10.2 |
| Influenza due to other identified influenza virus with other manifestations | J10.8 |
| Influenza due to other identified influenza virus with encephalopathy | J10.81 |
| Influenza due to other identified influenza virus with myocarditis | J10.82 |
| Influenza due to other identified influenza virus with otitis media | J10.83 |
| Influenza due to other identified influenza virus with other manifestations | J10.89 |
| Influenza due to unidentified influenza virus | J11 |
| Influenza due to unidentified influenza virus with pneumonia | J11.0 |
| Influenza due to unidentified influenza virus with unspecified type of pneumonia | J11.00 |
| Influenza due to unidentified influenza virus with specified pneumonia | J11.08 |
| Influenza due to unidentified influenza virus with other respiratory manifestations | J11.1 |
| Influenza due to unidentified influenza virus with gastrointestinal manifestations | J11.2 |
| Influenza due to unidentified influenza virus with other manifestations | J11.8 |
| Influenza due to unidentified influenza virus with encephalopathy | J11.81 |
| Influenza due to unidentified influenza virus with myocarditis | J11.82 |
| Influenza due to unidentified influenza with otitis media | J11.83 |
| Influenza due to unidentified influenza virus with other manifestations | J11.89 |
| Viral pneumonia, not elsewhere classified | J12 |
| Adenoviral pneumonia | J12.0 |
| Respiratory syncytial virus pneumonia | J12.1 |
| Parainfluenza virus pneumonia | J12.2 |
| Human metapneumovirus pneumonia | J12.3 |
| Other viral pneumonia | J12.8 |
| Pneumonia due to SARS-associated coronavirus | J12.81 |
| Pneumonia due to coronavirus disease 2019^c^ | J12.82 |
| Other viral pneumonia | J12.89 |
| Viral pneumonia, unspecified | J12.9 |
| Pneumonia due to Streptococcus pneumoniae | J13 |
| Pneumonia due to Hemophilus influenzae | J14 |
| Bacterial pneumonia, not elsewhere classified | J15 |
| Pneumonia due to Klebsiella pneumoniae | J15.0 |
| Pneumonia due to Pseudomonas | J15.1 |
| Pneumonia due to staphylococcus | J15.2 |
| Pneumonia due to staphylococcus, unspecified | J15.20 |
| Pneumonia due to staphylococcus aureus | J15.21 |
| Pneumonia due to Methicillin susceptible Staphylococcus aureus | J15.211 |
| Pneumonia due to Methicillin resistant Staphylococcus aureus | J15.212 |
| Pneumonia due to other staphylococcus | J15.29 |
| Pneumonia due to streptococcus, group B | J15.3 |
| Pneumonia due to other streptococci | J15.4 |
| Pneumonia due to Escherichia coli | J15.5 |
| Pneumonia due to other aerobic Gram-negative bacteria | J15.6 |
| Pneumonia due to Mycoplasma pneumoniae | J15.7 |
| Pneumonia due to other specified bacteria | J15.8 |
| Unspecified bacterial pneumonia | J15.9 |
| Pneumonia due to other infectious organisms, not elsewhere classified | J16 |
| Chlamydial pneumonia | J16.0 |
| Pneumonia due to other specified infectious organisms | J16.8 |
| Pneumonia in diseases classified elsewhere | J17 |
| Pneumonia, unspecified organism | J18 |
| Bronchopneumonia, unspecified organism | J18.0 |
| Lobar pneumonia, unspecified organism | J18.1 |
| Hypostatic pneumonia, unspecified organism | J18.2 |
| Other pneumonia, unspecified organism | J18.8 |
| Pneumonia, unspecified organism | J18.9 |
| Acute bronchitis | J20 |
| Acute bronchitis due to Mycoplasma pneumoniae | J20.0 |
| Acute bronchitis due to Hemophilus influenzae | J20.1 |
| Acute bronchitis due to streptococcus | J20.2 |
| Acute bronchitis due to coxsackievirus | J20.3 |
| Acute bronchitis due to parainfluenza virus | J20.4 |
| Acute bronchitis due to respiratory syncytial virus | J20.5 |
| Acute bronchitis due to rhinovirus | J20.6 |
| Acute bronchitis due to echovirus | J20.7 |
| Acute bronchitis due to other specified organisms | J20.8 |
| Acute bronchitis, unspecified | J20.9 |
| Acute bronchiolitis | J21 |
| Acute bronchiolitis due to respiratory syncytial virus | J21.0 |
| Acute bronchiolitis due to human metapneumovirus | J21.1 |
| Acute bronchiolitis due to other specified organisms | J21.8 |
| Acute bronchiolitis, unspecified | J21.9 |
| Unspecified acute lower respiratory infection | J22 |
| Other specified diseases of upper respiratory tract | J39.8 |
| Disease of upper respiratory tract, specified | J39.9 |
| Bronchitis, not specified as acute or chronic | J40 |
| Acute respiratory distress syndrome | J80 |
| Pulmonary edema | J81 |
| Acute pulmonary edema | J81.0 |
| Chronic pulmonary edema | J81.1 |
| Pleural effusion, not elsewhere classified | J90 |
| Acute respiratory failure | J96.0 |
| Acute respiratory failure, unspecified whether with hypoxia or hypercapnia | J96.00 |
| Acute respiratory failure with hypoxia | J96.01 |
| Acute respiratory failure with hypercapnia | J96.02 |
| Acute and chronic respiratory failure | J96.2 |
| Acute and chronic respiratory failure, unspecified whether with hypoxia or hypercapnia | J96.20 |
| Acute and chronic respiratory failure with hypoxia | J96.21 |
| Acute and chronic respiratory failure with hypercapnia | J96.22 |
| Respiratory failure, unspecified, unspecified | J96.9 |
| Respiratory failure, unspecified, unspecified whether with hypoxia or hypercapnia | J96.90 |
| Respiratory failure, unspecified with hypoxia | J96.91 |
| Respiratory failure, unspecified with hypercapnia | J96.92 |
| Other diseases of bronchus, not elsewhere classified | J98.09 |
| Pulmonary collapse | J98.1 |
| Atelectasis | J98.11 |
| Other pulmonary collapse | J98.19 |
| Other disorders of lung | J98.4 |
| Other specified respiratory disorders | J98.8 |
| Respiratory disorder, unspecified | J98.9 |
| Shock during or following labor and delivery | O75.1 |
| Pyrexia during labor, not elsewhere classified | O75.2 |
| Pyrexia of unknown origin following delivery | O86.4 |
| Other viral diseases complicating pregnancy/childbirth | O98.5 |
| Other viral diseases complicating pregnancy | O98.51 |
| Other viral diseases complicating pregnancy, first trimester | O98.511 |
| Other viral diseases complicating pregnancy, second trimester | O98.512 |
| Other viral diseases complicating pregnancy, third trimester | O98.513 |
| Other viral diseases complicating pregnancy, unspecified trimester | O98.519 |
| Other viral diseases complicating childbirth | O98.52 |
| Other viral diseases complicating the puerperium | O98.53 |
| Other maternal infectious and parasitic diseases complicating pregnancy | O98.81 |
| Other maternal infectious and parasitic diseases complicating pregnancy, first trimester | O98.811 |
| Other maternal infectious and parasitic diseases complicating pregnancy, second trimester | O98.812 |
| Other maternal infectious and parasitic diseases complicating pregnancy, third trimester | O98.813 |
| Other maternal infectious and parasitic diseases complicating pregnancy, unspecified trimester | O98.819 |
| Diseases of the respiratory system complicating pregnancy | O99.51 |
| Diseases of the respiratory system complicating pregnancy, first trimester | O99.511 |
| Diseases of the respiratory system complicating pregnancy, second trimester | O99.512 |
| Diseases of the respiratory system complicating pregnancy, third trimester | O99.513 |
| Diseases of the respiratory system complicating pregnancy, unspecified trimester | O99.519 |
| Hemoptysis | R04.2 |
| Cough | R05 |
| Acute cough | R05.1 |
| Subacute cough | R05.2 |
| Chronic cough | R05.3 |
| Cough syncope | R05.4 |
| Other specified cough | R05.8 |
| Cough, unspecified | R05.9 |
| Dyspnea | R06.0 |
| Dyspnea, unspecified | R06.00 |
| Orthopnea | R06.01 |
| Shortness of breath | R06.02 |
| Acute respiratory distress | R06.03 |
| Other forms of dyspnea | R06.09 |
| Stridor | R06.1 |
| Wheezing | R06.2 |
| Tachypnea, not elsewhere classified | R06.82 |
| Chest pain on breathing | R07.1 |
| Asphyxia and hypoxemia | R09.0 |
| Asphyxia | R09.01 |
| Hypoxemia | R09.02 |
| Respiratory arrest | R09.2 |
| Abnormal sputum | R09.3 |
| Nasal congestion | R09.81 |
| Other specified symptoms and signs involving the circulatory and respiratory systems | R09.89 |
| Fever of other and unknown origin^d^ | R50 |
| Drug induced fever | R50.2 |
| Other specified fever | R50.8 |
| Fever presenting with conditions classified elsewhere | R50.81 |
| Postprocedural fever | R50.82 |
| Postvaccination fever | R50.83 |
| Febrile nonhemolytic transfusion reaction | R50.84 |
| Fever, unspecified | R50.9 |
| Shock, unspecified | R57.9 |
| Severe sepsis with septic shock | R65.21 |
| Chills (without fever) | R68.83 |
| COVID-19, virus identified^c^ | U07.1 |
| COVID-19, virus not identified^c^ | U07.2 |

COVID-19, coronavirus disease 2019; SARS, severe acute respiratory syndrome.

^a^ Thompson MG, Kwong JC, Regan AK, Katz MA, et al. Influenza vaccine effectiveness in preventing influenza-associated hospitalizations during pregnancy: a multi-country retrospective test negative design study, 2010–2016. Clin Infect Dis. 2019;68:1444-1453.

^b^ Gray shading used to separate groups of codes (e.g., J01.x codes vs J02.x codes).

^c^ Code was not in code set used in Thompson et al. (2019). Added to account for COVID-19.

^d^ Patients were also considered to have ARFI if their record included a temperature reading of >100.4°F.

Table S3. CVX, CPT, and NDC Codes for 2022–2023 influenza vaccines

| **Influenza Vaccine Type** | **CVX codes** | **CPT Codes** | **NDC Codes** |
| --- | --- | --- | --- |
| QIVe | 150, 158 | 90686, 90687, 90688 | 19515-0808-52, 19515-0808-41, 33332-0422-10, 33332-0422-11, 33332-0322-04, 33332-0322-03, 49281-0422-88, 49281-0422-50, 49281-0422-10, 49281-0422-58, 49281-0637-15, 49281-0637-78, 58160-0890-41, 58160-0890-52 |
| QIVc | 171, 186 | 90674,  90756 | 70461-0322-03, 70461-0322-04, 70461-0422-10, 70461-0422-11 |

CPT, current procedural terminology; CVX, vaccine administered code set; NDC, National Drug Code; QIVc, cell-based quadrivalent influenza vaccine; QIVe, egg-based quadrivalent influenza vaccine.

Influenza Test Data Mapping

Influenza test results were reported in both structured and unstructured form. Structured results refer to a SNOMED concept and were mapped to identify test results as shown in the table below:

| **SNOMED Concept** | **SNOMED Description** | **Assigned Category** |
| --- | --- | --- |
| 260385009 | Negative | Negative / Not Detected |
| 260415000 | Not detected | Negative / Not Detected |
| 260373001 | Detected | Positive / Detected |
| 10828004 | Positive | Positive / Detected |

Unstructured, or non-standardized, results are short phrases sent from the lab or entered in the EHR to describe the result. The unstructured data is mapped to categories of ‘Negative / Not Detected’ and ‘Positive / Detected’ for the purposes of this analysis using the following process:

1. First, entries that have both the terms ‘pos’ and ‘neg’ in them are mapped these to ‘Positive and Negative’ – for the purposes of these analyses, these terms will be considered Positive findings, as at least one flu type was marked to be positive.
   1. Using the results of the feasibility analysis, the most common unstructured values that are mapped to this term are: pos a / neg b, a- positive b- negative, neg a / pos b, a - positive b – negative. They comprise 0.4% of all results and 36.3% of all results mapped to ‘Positive and Negative’
2. Next, remaining entries that have either the term ‘neg’ or ‘not detected’ are mapped to ‘Negative / Not Detected’
   1. Using the results of the feasibility analysis, the most common unstructured values that are mapped to this term are: negative, not detected, neg, negative for influenza a and b. They comprise 55.5% of all results and 87.3% of all results mapped to ‘Negative / Not Detected’
3. Then, remaining entries that have either the term ‘pos’ or ‘detected’ are mapped to ‘Positive / Detected’
   1. Using the results of the feasibility analysis, the most common unstructured values that are mapped to this term are: positive, detected, pos, positive a. They comprise 9.9% of all results and 83.2% of all results mapped to ‘Positive / Detected’
4. Finally, the remaining entries are categorized as unmapped and cannot be used for analysis
   1. Using the results of the feasibility analysis, the most common unstructured values that are mapped to this term are: xyxyxy, pass, yes, present. They comprise 10.3% of all results and 65.4% of all unmapped non-numeric results.
   2. Numeric values from the unstructured data are also considered unmapped and not usable for this analysis. They comprise 7.5% of all results.

There were approximately 3,500 possible values in the Veradigm unstructured result field representing approximately 1.9 million results, 1.6 million of which were deemed to be usable as positive or negative. Some tests had multiple results, which is why the 1.6 million relates to the smaller distinct test count, with a usable result of 1.45 million. The top 669 values, which represented 99% of all results, were reviewed manually to ensure that the mapping described above was accurate.

Outcome Definitions

A case was defined by a positive influenza test result within the season timeframe ascertained using CPT or LOINC codes (Supplementary Tables 1 and 3). Case definitions were explored and defined in the feasibility assessment preceding the 2017–2020 study. Influenza test results are reported in both structured and unstructured form in the data. Structured results refer to a SNOMED concept (Supplementary Materials, Influenza Test Data Mapping). Unstructured, or non-standardized, results are short phrases sent from the lab or entered in the EHR to describe the result. Influenza test results from the structured and unstructured data were classified as ‘Negative / Not Detected’ or ‘Positive / Detected’.

Regarding the date of a test, if a test result had an available ordered date for the test, that date was used as the Test Index Date; if the ordered date was missing, the reported date of the test was used as the Test Index Date, and if that was missing, the date the record was created in the EHR was used as the Test Index Date.

If a patient had multiple test results in a given season, the following logic was implemented to select the relevant test result:

- If the patient had both positive and negative test results in a season, the only or earliest positive influenza test result was evaluated, and the patient was assigned as a case.
- Else if the patient had no negative influenza test results and more than one positive influenza test result in a season, the earliest positive influenza test result was evaluated, and the patient was assigned as a case.
- Else if the patient had no positive influenza test results and one or more negative results in a season, the only or earliest negative influenza test was evaluated, and the patient was assigned as a control.

Hence, each patient was assigned one status, either case or control, and controls had to have tested negative through the end of the influenza season (i.e., have no positive results). The figure below shows four synthetic patient event histories to help illustrate how case assignment was performed. Patient 1 would be assigned to the case population because they had an ARFI during the influenza season within +/- 7 days of their first positive influenza test and at least 14 days after their vaccination. (Of note, 14 days are required to allow for the development of vaccine-specific antibodies following the date of recorded vaccination.) In this case, the prior influenza test does not impact case assignment because it was a negative test. Patient 2 would be excluded from the study because their first positive test came before their vaccination. Patient 3 would be excluded from the study because their first positive test was not within ±7 days of an ARFI. The earlier negative test would not be evaluated because the patient had a positive test during the season. Patient 4 would be excluded from the study because they had a positive influenza test during the test-positive exclusion period, May 22, 2022 to October 2, 2022.

Figure. Case Assignment and Exclusion Examples


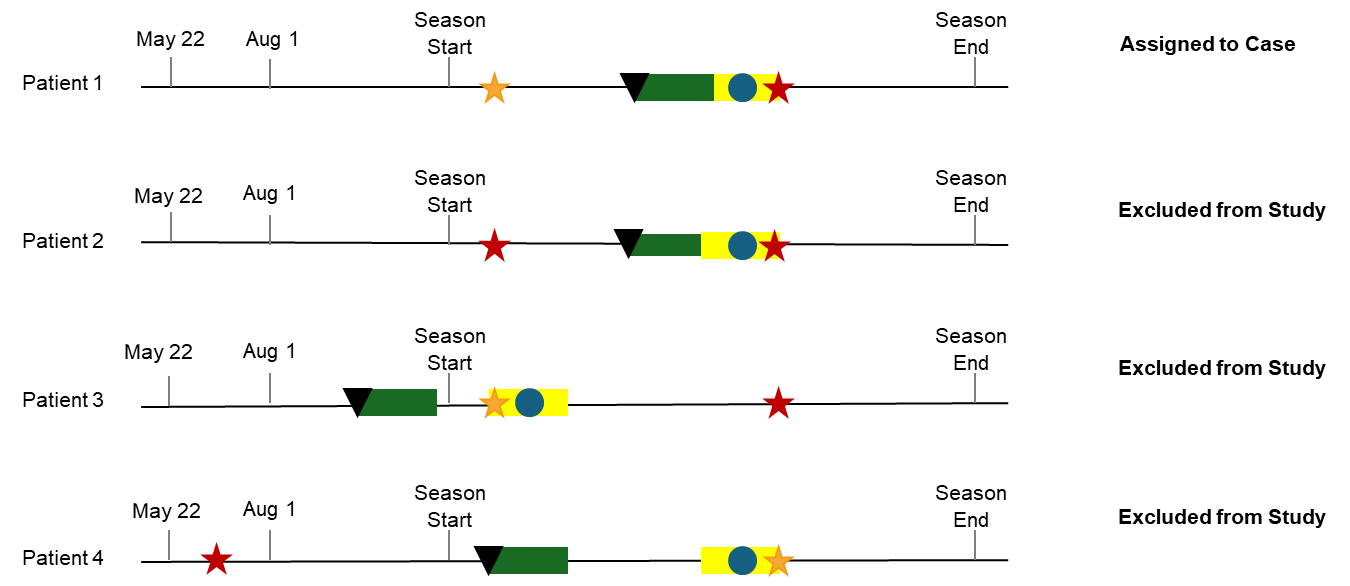


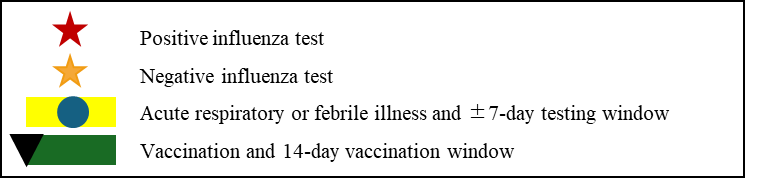


Molecular tests (e.g., PCR tests) and antigen tests were included, and type of test was recorded as a covariate to enable consideration of subgroup analysis by test type if feasible. Antibody and culture tests were excluded to avoid potential bias due to the lack of specificity for antibody tests to reliably detect acute disease and potential differences in patients tested by culture methods.

Statistical Analysis

*Variables*

The following variables were captured at Test Index Date, Vaccination Date or in the Pre-Vaccination Period for patients included in this study:

- Age at Date of Vaccination (continuous, categorical: 0-4, 5-17, 18-49, 50-64). Defined as spline function in adjusted analysis, as described below
- Sex (Female, Male)
- Race (Asian, Black or African American, White, Other, Not Reported). Race was a non-required self-reported field in the EHRs. When patients chose not to disclose this information or providers chose to not enter it, race was coded as “not reported”
- Ethnicity (Hispanic, Non-Hispanic, Not Reported). Ethnicity was a non-required self-reported field in the EHRs. When patients chose not to disclose this information or providers chose not to enter it, ethnicity was coded as “not reported”
- Test Index Date (calendar days since the start of influenza season). In descriptive tables shown as month of Index Date and graphed as number of weeks since the start of influenza season. Defined as a spline function in adjusted analysis, as described below
- Week of Vaccination Date (number of weeks since the start of the Vaccination Intake Timeframe)
- Insurance/Payer at Test Index Date (Commercial, Medicaid, Medicare, Unknown/Other)
- Geographic Region (Region 1-Region 10). Geographic region was categorized according to the definition from the U.S. Department of Health and Human Services (HHS). For patients with missing region in the EHR it was defined in the claims portion of the Integrated Dataset (see Supplementary Table 5 for a list of states in each HHS Region)
- Test Type (Antigen, Molecular)
- COVID-19 Vaccination Receipt during the 6 months prior to the Test Index Date (yes/no)
- Charlson Comorbidity Index (CCI)^1^ from the Pre-Vaccination Period
- Underlying conditions identified by the CDC as posing higher risk of complications due to influenza^2,3^
  - Asthma
  - Neurologic and neurodevelopmental conditions
  - Blood disorders (such as sickle cell disease)
  - Chronic lung disease (such as chronic obstructive pulmonary disease [COPD] and cystic fibrosis)
  - Endocrine disorders (such as diabetes mellitus)
  - Heart disease and related conditions (such as congenital heart disease, congestive heart failure and coronary artery disease)
  - Kidney diseases
  - Liver disorders
  - Metabolic disorders (such as inherited metabolic disorders and mitochondrial disorders)
  - People with obesity with a body mass index (BMI) of 40 or higher
  - People with a weakened immune system due to disease (such as people with human immunodeficiency virus/acquired immunodeficiency syndrome or some cancers such as leukemia) or medications (such as those receiving chemotherapy or radiation treatment for cancer, or persons with chronic conditions requiring chronic corticosteroids or other drugs that suppress the immune system)
  - People who have had a stroke
- Healthcare resource utilization defined by three variables:
  - Number of outpatient visits (all-cause) (continuous)
  - Number of inpatient admissions (all-cause) (categorical: 0, 1, ≥2)
  - Number of emergency department visits (all-cause) (categorical: 0, 1, ≥2)

*Descriptive Analysis*

Summary tables of patient baseline demographic and clinical characteristics were reported by vaccine type (QIVc, QIVe), by case status (influenza test-positive vs influenza test-negative), and by both case status and vaccine type (cases with QIVc, controls with QIVc, cases with QIVe, controls with QIVe). Categorical variables were presented as counts and percentages, continuous variables were presented by mean, standard deviation (SD), median, and interquartile range (IQR). The proportion of patients with missing or unknown, or undocumented values for race and ethnicity were also reported. The percentage of patients with Test Index Dates in each week of the influenza season was summarized by vaccine type and by case status in descriptive tables and presented as figures. Vaccine exposure group balance for covariates among the controls were assessed using Austin’s standardized mean difference (SMD).^4^

*Covariate Balance—Standardized Mean Differences*

In a test-negative design study, the control group is intended to represent the overall source population. It is within this control group that characteristics of patients in each vaccine group are evaluated for potential confounding.^5^ For this study, covariate balance between vaccine exposure group among the controls was assessed using SMDs. An SMD with an absolute value ≤ 0.1 was used to indicate a negligible difference in proportions between the groups.^4^ SMDs were generated to assess differences in each variable listed between the QIVc and QIVe controls prior to any statistical adjustment or weighting.

The SMD compares the difference in means in units of the pooled standard deviation. Unlike other statistical tests of hypothesis, the SMD is not influenced by sample size. Thus, the use of the SMD can be used to compare the balance in measured variables between the QIVc- and QIVe-exposed controls in the unweighted sample with balance in a weighted sample.

For continuous variables, the SMD is defined as:

$$d= \frac{(\bar{x}_{group 1}- \bar{x}_{group 0})}{\sqrt{\frac{s_{group 1}^{2}+ s_{group 0}^{2}}{2}}}$$

where $\bar{x}_{group 1}$ and $\bar{x}_{group 0}$ denote the sample means of the covariate in the QIVc and QIVe exposure groups among controls (or case and control group for the univariate comparison), while $s_{group 1}^{2}$and $s_{group 0}^{2}$ denote the sample variances of the covariate in the QIVc and QIVe group (or case and control group).

For categorical variables, the SMD is defined as:

$$d= \frac{(\hat{p}_{group 1-}\hat{p}_{group 0})}{\sqrt{\frac{\hat{p}_{group 1}\left( 1- \hat{p}_{group 1} \right)+ \hat{p}_{group 0}\left( 1- \hat{p}_{group 0} \right)}{2}}}$$

where $\hat{p}_{group 1}$ and $\hat{p}_{group 0}$ denote the prevalence or means of the categorical variable in the QIVc and QIVe exposure group among the controls (or case and control group).

*Primary Analyses*

The primary analysis assessed the relative vaccine effectiveness (rVE) between QIVc and QIVe using a retrospective test-negative design. With this design, the same criteria for clinical presentation is used for selection of both cases and controls, minimizing the likelihood for selection bias in observational studies.^6,7^ This principle was applied in the current study by restricting to vaccinated patients with a documented ARFI within 7 days (before or after) of the influenza test during the influenza season.

The rVE compared the odds of testing positive for influenza among QIVc vs. QIVe recipients. A generalized linear model with a logit link was used to obtain odds ratios (ORs) comparing influenza-positive cases and influenza-negative controls, with vaccine type received as the exposure of interest. The rVE was calculated as rVE = (1 ‒ OR_adjusted_) × 100 and reported with 95% confidence intervals (CI). The primary analysis applied a doubly robust approach, combining inverse probability of treatment weighting (IPTW) and multivariable adjustment. Unadjusted rVEs were also reported.

*Covariate Transformation*

The primary analysis adjusted for calendar time (in days) between the start of the influenza season and the Test Index Date to capture variation in the risk of infection over time and potential waning of vaccine effectiveness as the season progressed. Because it was assumed that the confounding effects of time on vaccination and influenza test result were not linear, the variable for time from start of the influenza season to Test Index Date was transformed using a spline function to summarize the non-linear relationship between elapsed time and influenza test result.^8^ The approach used a restricted cubic spline to split the range of values of elapsed time into intervals using ‘knots’ that define where one interval ends, and another begins. Prior to inclusion in the adjusted analysis, the optimal number (e.g., 3, 4, or 5) and placement (e.g., at quartiles, quintiles, etc.) of knots was determined using the Akaike information criteria (AIC).^8^ In this study, the calendar time (in days) covariate was transformed into a spline with 5 knots placed at days 51, 69, 89, 118, and 159 (AIC = 36,902.877).

The age covariate within the model also underwent transformation into a spline variable to better capture its non-linear influence on the risk of infection and vaccine effectiveness and to allow smoother transition between age groups. Knot placements were assessed using either a data driven approach (similar to the approach which was used for calendar time (in days)), or *a priori* knot placement aligned with key age groups for vaccine recommendations, specifically 5, 18, and 50 years. The spline models with varying number of knots (e.g., 1, 2 or 3) and placements were fitted, and the optimal number and placement of knots was determined based on the spline model with the lowest AIC. In this study, the age covariate was transformed into a spline with 5 knots placed at ages 4, 10, 18, 40, and 55 years (AIC = 38,696.268).

*Weighting Using Inverse Probability Treatment Weighting*

Stabilized IPTWs were used to account for the *a priori* defined covariates age (as spline), sex, region, Test Index Date (as spline), COVID-19 vaccination history, and any additional covariate with an SMD with an absolute value > 0.1. IPTW allowed maximal use of available data but may be distorted by cases with very large or very small propensity scores, which may result in very small or large IPTW values. Stabilized weighting avoids the effect of extreme weights on the variability of the estimated treatment effect.

The methodology used to perform stabilized IPTW is as follows:

1. Among the influenza-test-negative controls, logistic regression models predictive of treatment group membership (i.e., QIVc vs. QIVe) provided propensity scores pj based on the study covariates. The models included the *a priori* covariates listed above and additional covariates identified as having a non-negligible difference (imbalanced) between vaccine groups among the controls.
2. These fitted models were then applied to the influenza-positive cases to calculate propensity of treatment group membership scores for the cases (pj).
3. Next, both cases and controls in each of the vaccine groups were assigned a stabilized weight as follows:
   1. IPTW values wj were calculated as wj = 1 / (pj) for the QIVc group and wj = 1 / (1-pj) for the QIVe group.
   2. Stabilized weights w*j were obtained by multiplying IPTW values by the proportion of individuals in the QIVc and QIVe groups, respectively. For the QIVc group w*j = pt * wj, while for the QIVe group w*j = (1- pt) * wj where pt = Nt / (Nt+Nc), the proportion of individuals in the QIVc group.
4. An absolute standardized difference value was calculated for all covariates (categorical as well as continuous variables). The absolute standardized difference compares the difference in means of covariates measured in standard deviation units. The absolute standardized differences in the unweighted and weighted covariates between QIVc and QIVe is displayed graphically.

*Primary Analysis (Doubly Robust)*

The primary analysis used a doubly robust approach to adjust for any potential confounding (34). The IPTW sample was used in a multivariable model with age (as spline), sex, region, Test Index Date (as spline), COVID-19 vaccination history, and any other covariates that remained imbalanced following IPTW.

| Estimate π=Pr(influenza positive=1\|x) for logit(π)= α + β’x  where each observation is appropriately IPTW weighted and x is a multi-item vector of vaccine type (QIVc vs. QIVe), *a priori* defined covariates and remaining imbalanced study covariates |
| --- |

*Sensitivity Analyses*

Three prespecified sensitivity analyses were conducted to evaluate the robustness of key assumptions and residual confounding in the main analysis.

Propensity to be Tested

This sensitivity analysis addressed the potential bias of which patients are given an influenza test. The analysis assessed all vaccinated (with QIVc or QIVe) patients with a documented ARFI during the influenza season, for whom we calculated the propensity to be tested during the influenza season. Demographic (age as a continuous variable, sex, race, ethnicity, region, insurance information) and clinical (CCI score, COVID-19 vaccination history, high risk conditions, healthcare utilization, and week of vaccination) covariates were used to estimate the propensity to be tested and propensity-for-testing estimators were created using the inverse of the score. The primary analysis was then repeated, accounting for the propensity-for-testing scores as a covariate in the model.

Matching on Week of Test Index Date

In this sensitivity analysis, rather than adjusting for time from influenza season start date to Test Index Date using a spline, cases were matched with up to 5 controls on the exact week of the Test Index Date (test week). The intent was to further examine if there was confounding associated with the timing of the test via a matched analysis since this is less sensitive to model misspecification.^9,10^ A greedy nearest neighbor approach was used to match cases to controls by test week only. To optimize balance, we used variable ratio matching and capped the number of matched controls at five. This approach was selected because it outperforms fixed-ratio matching in terms of bias, precision, and mean squared error (MSE), while limiting the potential for bias.^11,12^ The subset of patients used in this analysis was referred to as the Matched on Week Population. After matching on calendar week, doubly robust IPTW and multivariable conditional logistic regression methodology was applied stratifying the analysis by . matched risk set. The model excluded the spline function for Test Index Date covariate.

Seasonal Peak Period

This sensitivity analysis limited the subset of the population in the primary analysis to those whose Test Index Date fell within the peak period of the influenza season to examine potential bias (underestimation of rVE) from lower predictive ability of influenza tests when influenza activity is low. Limiting Test Index Dates to a period of higher test positivity, decreases the possibility of case misclassification during periods of low influenza activity.^13,14^

The seasonal peak period cut points were November 6, 2022 (week 45) through December 24, 2022 (week 51), as determined using the moving epidemic method (MEM),^^[[1]](#footnote-1)^^ based on CDC data reporting the percentage of outpatient influenza tests that were positive.^15-17^ Specifically, data from the 2003-2004 through 2015-2016 influenza seasons were used to establish a baseline, with a season start cut of 14.47% positive and season end cut of 17.48% positive. The MEM—a standard approach for establishing epidemic thresholds using historical epidemiological data—is widely applied in Europe and has also been used in recent research in the U.S. assessing the rVE of influenza vaccines.^18^

For this sensitivity analysis, the sample was re-weighted using IPTW and the model was re-specified, following the same procedure described for the primary analysis.

*Missing Values*

Among demographic variables, missing values were captured as a category—i.e., as “Missing” or “Not Reported.” Missing values were not imputed. Patients with missing values for age, sex, or geography were excluded from the study.

For the continuous variable CCI, patients were assigned a score of 0 if they had no record of any of the conditions used for the CCI index.

Table S4. Summary of statistical analyses

| **Analysis** | **Description** | **Weighting variables** | **Multivariable model covariates** | | **Final covariates in adjusted statistical models** | |
| --- | --- | --- | --- | --- | --- | --- |
|  |  |  |  |  | **IPTW** | **Doubly robust (IPTW and multivariable)** |
| **Descriptive Analysis** | |  | |  | | |
| Patient Demographic and Clinical Characteristics | Descriptive statistics for covariates by case status, vaccine type, and case status by vaccine type; SMDs | N/A | N/A | | N/A | N/A |
| **Unadjusted Analysis** | |  | |  | | |
| Unadjusted rVEs and 95% CIs | rVE, unweighted, no covariates | N/A | N/A | | N/A | N/A |
| **Primary Analysis** | |  | |  | | |
| Doubly Robust Analysis: rVEs and 95% CIs adjusted using IPTW and multivariable regression modeling | rVE, weighted, covariate adjusted | Age (as spline), sex, region, Test Index Date* (as spline), COVID-19 vaccination history, and any other covariate with \|SMD \| > 0.1 | Age (as spline), sex, region, Test Index Date* (as spline), COVID-19 vaccination history and, any other covariates that are imbalanced after weighting, i.e., \|weighted SMD\| > 0.1**Error! Reference source not found.** | | A priori: Age (as spline), Sex, Region, Test Index Date (as spline), COVID Vaccination History  Based on SMD: ethnicity, week of vaccination, payer, mean CCI score, neurologic and neurodevelopmental conditions, endocrine disorders, heart disease and related conditions, metabolic disorders | IPT weight  A priori: Age (as spline), Sex, Region, Test Date (as spline), COVID Vaccination History  Based on SMD: Test type |
| **Sensitivity Analyses** | |  | |  | | |
| 1. Doubly Robust Analysis including propensity-to-be-tested scores as a covariate | rVE, weighted, covariate adjusted | Age (as spline), sex, region, Test Index Date* (as spline), COVID-19 vaccination history, and any other covariate with \|SMD \| > 0.1 | Age (as spline), sex, region, Test Index Date* (as spline), COVID-19 vaccination history, propensity to be tested estimator, and any other covariates that are imbalanced after weighting, i.e., \|weighted SMD\| > 0.1**Error! Reference source not found.** | | A priori: Age (as spline), Sex, Region, Test Index Date spline, COVID Vaccination History  Based on SMD: ethnicity, week of vaccination, payer, mean CCI score, neurologic and neurodevelopmental conditions, endocrine disorders, heart disease and related conditions, metabolic disorders | IPT weight  A priori: Age (as spline), Sex, Region, Test Index Date (as spline), COVID Vaccination History, Propensity to be Tested Estimator  Based on SMD: Test type |
| 2. Doubly Robust Analysis including exact matching on the week of the Test Index Date | rVE, weighted, covariate adjusted | Age (as spline), sex, region, COVID-19 vaccination history, and any other covariate with \|SMD \| > 0.1 | Age (as spline), sex, region, COVID-19 vaccination history, and any other covariates that are imbalanced after weighting, i.e., \|weighted SMD\| > 0.1**Error! Reference source not found.** | | A priori: Age (as spline), Sex, Region, COVID Vaccination History  Based on SMD: race, ethnicity, week of vaccination, payer, mean CCI score, neurologic and neurodevelopmental conditions, endocrine disorders, heart disease and related conditions, metabolic disorders, liver disorders | IPT weight  A priori: Age (as spline), Sex, Region, COVID Vaccination History  Based on SMD: Test type |
| 3. Doubly Robust Analysis using seasonal peak period | rVE, weighted, covariate adjusted | Age (as spline), sex, region, Test Index Date* (as spline), COVID-19 vaccination history, and any other covariate with \|SMD \| > 0.1 | Age (as spline), sex, region, Test Index Date* (as spline), COVID-19 vaccination history, and any other covariates that are imbalanced after weighting, i.e., \|weighted SMD\| > 0.1**Error! Reference source not found.** | | A priori: Age (as spline), Sex, Region, Test Index Date (as spline), COVID Vaccination History  Based on SMD: race, ethnicity, week of vaccination, payer, neurologic and neurodevelopmental conditions, endocrine disorders, heart disease and related conditions, metabolic disorders | IPT weight  A priori: Age (as spline), Sex, Region, Test Index Date (as spline), COVID Vaccination History  Based on SMD: Test type |

CI, confidence interval; CCI, Charlson comorbidity index; IPTW, inverse probability of treatment weighting; rVE, relative vaccine effectiveness; SMD, standardized mean difference

* “Test Index Date” refers to the calendar time between the start of the influenza season and the Test Index Date.

Table S5. HHS regions.

| **Region** | **States** |
| --- | --- |
| 1 | Connecticut, Maine, Massachusetts, New Hampshire, Rhode Island, Vermont |
| 2 | New Jersey, New York, Puerto Rico, the Virgin Islands |
| 3 | Delaware, District of Columbia, Maryland, Pennsylvania Virginia, West Virginia |
| 4 | Alabama, Florida, Georgia, Kentucky, Mississippi, North Carolina, South Carolina, Tennessee |
| 5 | Illinois, Indiana, Michigan, Minnesota, Ohio, Wisconsin |
| 6 | Arkansas, Louisiana, New Mexico, Oklahoma, Texas |
| 7 | Iowa, Kansas, Missouri, Nebraska |
| 8 | Colorado, Montana, North Dakota, South Dakota, Utah, Wyoming |
| 9 | Arizona, California, Hawaii, Nevada, American Samoa, Commonwealth of the Norther Mariana Islands, Federated States of Micronesia, Guam, Marshall Islands, Republic of Palau |
| 10 | Alaska, Idaho, Oregon, Washington |

HHS, U.S. Health and Human Services.

Table S6. Model input data for analysis of influenza burden averted

|  | **0–4 years** | **5–17 years** | **18–49 years** | **50–64 years** | **Source** |
| --- | --- | --- | --- | --- | --- |
| **Vaccine data** | | | | | |
| aVE QIVe, % (95% CI)^a^ | 48 (44–52) | | 45 (41–48) | | CDC^19^ |
| rVE QIVc vs QIVe, % (95% CI)^b^ | 7.7 (0.9–13.9) | | | |  |
| Vaccine coverage, % ± 95% CI^c^ | 65.6 ± 1.1 | 55.1 ± 0.7 | 35.2 ± 0.6 | 50.1 ± 1.0 | CDC^20^ |
| **Influenza burden and healthcare resource use without vaccination, n (95% CI)** | | | | | |
| Symptomatic cases | 3,342,052 (2,442,627–7,459,809) | 9,016,337 (6,297,171–23,573,171) | 11,162,710 (8,156,800–20,188,410) | 6,280,920 (4,687,388–11,331,457) | CDC^21^ |
| Outpatient visits | 2,239,175 (1,605,127– 5,046,109) | 4,688,495 (3,206,427– 12,265,167) | 4,130,203 (2,912,415– 7,564,222) | 2,700,796 (1,930,896– 4,978,886) | CDC^21^ |
| Hospitalizations | 23,299 (17,029– 52,006) | 24,722 (17,266– 64,635) | 62,656 (45,784– 113,318) | 66,607 (49,708– 120,167) | CDC^21^ |
| ICU admissions^d^ | 3,541 | 3,758 | 9,524 | 10,124 | CDC^22^ |
| Deaths | 228 (33–399) | 248 (84–696) | 1,003 (617–2,299) | 4,523 (2,795–8,853) | CDC^21^ |

aVE, absolute vaccine effectiveness; CI, confidence interval; ICU, intensive care unit; QIVe, egg-based cell-based quadrivalent influenza vaccine.

^a^Data from the Virtual SARS-CoV-2, Influenza, and Other respiratory viruses Network (VISION) outpatient network.

^b^rVE as reported in the present study.

^c^Percentage vaccinated. Percentages are weighted to the U.S. population. Month of vaccination was imputed for respondents with missing month of vaccination data. CIs are half-widths.

^d^ICU admission rate in the 2022–2023 season was 15.2%.

Figure S1. Subject selection

| 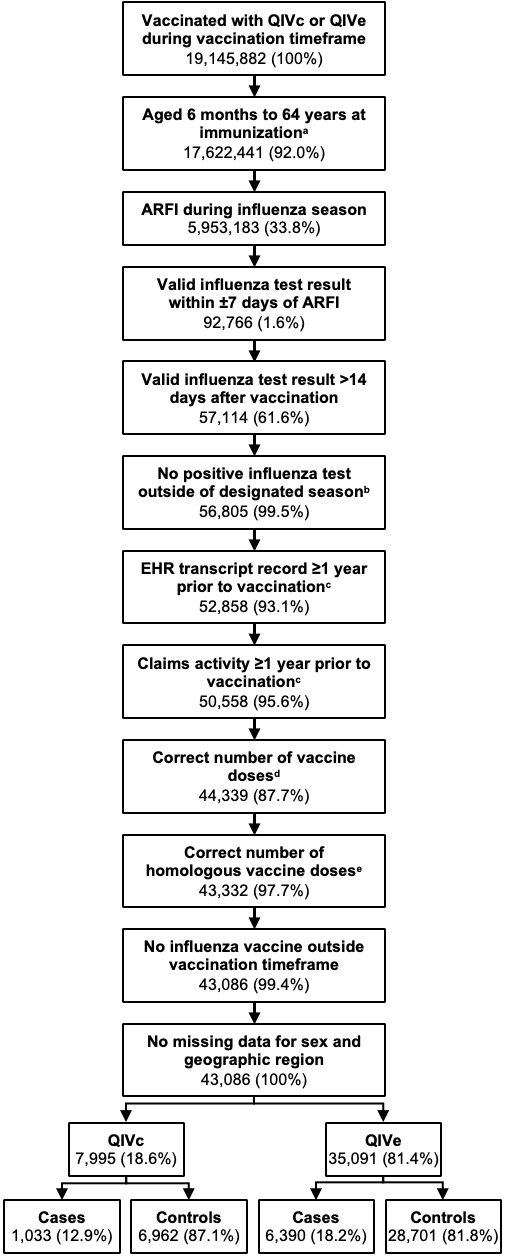 | Percentages shown for each cohort in the flow chart reflect the proportion of study subjects retained from the preceding cohort.  ^a^If a patient receives more than 1 vaccination, age at date of vaccination will be calculated based on the last vaccination during the vaccination intake timeframe (closest date to the test index date).  ^b^Subject does not have a positive influenza test between the end of the 2021–2022 influenza season (May 22, 2022) and the start of the 2022–2023 influenza season (October 2, 2022).  ^c^For subjects aged >1 year, subject has a transcript record in the Veradigm EHR or Komodo claims data at least 12 months prior to date of vaccination. For subjects age ≤1 year, subject has a transcript record in the Veradigm EHR or Komodo claims data at least 6 months prior to date of vaccination.  ^d^Subjects aged ≥9 years do not have more than 1 influenza immunization between the start date of the vaccination intake timeframe and the test index date. Subjects aged <9 years do not have (a) >2 influenza vaccine administrations or (b) 2 heterologous vaccine administrations between the start date of the vaccination intake timeframe and the test index date.  ^e^For subjects aged <9 years, subject has either 2 homologous QIVc or QIVe vaccine administrations at least 28 days apart and within the vaccination intake timeframe or at least one influenza vaccination administration on or prior to May 21, 2022.  ARFI, acute respiratory or febrile illness; aTIV, adjuvanted trivalent influenza vaccine; EHR, electronic health record; HD-TIV, high-dose trivalent influenza vaccine; LAIV, live attenuated influenza vaccine; QIVc, cell-based quadrivalent influenza vaccines; QIVe, egg-based quadrivalent influenza vaccines; rQIV, recombinant quadrivalent influenza vaccine; TIVe, egg-based trivalent influenza vaccine. |
| --- | --- |

**Table S7. Demographic and clinical characteristics of the overall study population**

| **Variable** | **Overall**  **(N=43,086)** | **QIVc** | | | **QIVe** | | |
| --- | --- | --- | --- | --- | --- | --- | --- |
|  |  | **Total (n=7,995)** | **Case**  **(n=1,033)** | **Control^a^**  **(n=6,962)** | **Total (n=35,091)** | **Case**  **(n=6,390)** | **Control^a^**  **(n=28,701)** |
| **Age at index date, years, mean (SD)** | 26.8 (21.8) | 37.9 (19.5) | 32.1 (21.0) | **38.7 (19.2)** | 24.3 (21.5) | 18.9 (19.1) | **25.5 (21.8)** |
| Age at index date, years, median (IQR) | 18 (42) | 42 (36) | 33 (42) | 43 (33) | 15 (39) | 11 (24) | 16 (41) |
| **Age group at index date, n (%)** |  |  |  |  |  |  |  |
| 0-4 years | 7,491 (17) | 323 (4) | 39 (4) | **284 (4)** | 7,168 (20) | 1,263 (20) | **5,905 (21)** |
| 5-17 years | 13,798 (32) | 1,524 (19) | 383 (37) | **1,141 (16)** | 12,274 (35) | 3,233 (51) | **9,041 (32)** |
| 18-49 years | 11,468 (27) | 3,198 (40) | 294 (28) | **2,904 (42)** | 8,270 (24) | 1,017 (16) | **7,253 (25)** |
| 50-64 years | 10,329 (24) | 2,950 (37) | 317 (31) | **2,633 (38)** | 7,379 (21) | 877 (14) | **6,502 (23)** |
| **Sex, n (%)** |  |  |  |  |  |  |  |
| Female | 25,333 (59) | 4,975 (62) | 580 (56) | 4,395 (63) | 20,358 (58) | 3,424 (54) | 16,934 (59) |
| Male | 17,753 (41) | 3,020 (38) | 453 (44) | 2,567 (37) | 14,733 (42) | 2,966 (46) | 11,767 (41) |
| **Race, n (%)** |  |  |  |  |  |  |  |
| White | 30,160 (70) | 5,825 (73) | 772 (75) | 5,053 (73) | 24,335 (69) | 4,448 (70) | 19,887 (69) |
| Black | 3,222 (7) | 550 (7) | 58 (6) | 492 (7) | 2,672 (8) | 453 (7) | 2,219 (8) |
| Asian | 1,311 (3) | 356 (4) | 48 (5) | 308 (4) | 955 (3) | 170 (3) | 785 (3) |
| Other | 2,772 (6) | 440 (6) | 55 (5) | 385 (6) | 2,332 (7) | 399 (6) | 1,933 (7) |
| Unknown/not reported | 5,621 (13) | 824 (10) | 100 (10) | 724 (10) | 4,797 (14) | 920 (14) | 3,877 (14) |
| **Ethnicity, n (%)** |  |  |  |  |  |  |  |
| Hispanic | 3,720 (9) | 516 (6) | 53 (5) | 463 (7) | 3,204 (9) | 656 (10) | 2,548 (9) |
| Non-Hispanic | 30,559 (71) | 6,115 (76) | 785 (76) | **5,330 (77)** | 24,444 (70) | 4,415 (69) | **20,029 (70)** |
| Unknown/not reported | 8,807 (20) | 1,364 (17) | 195 (19) | **1,169 (17)** | 7,443 (21) | 1,319 (21) | **6,124 (21)** |
| **HHS region, n (%)** |  |  |  |  |  |  |  |
| Region 1 | 2,792 (6) | 333 (4) | 59 (6) | **274 (4)** | 2,459 (7) | 588 (9) | **1,871 (7)** |
| Region 2 | 4,265 (10) | 731 (9) | 82 (8) | 649 (9) | 3,534 (10) | 697 (11) | 2,837 (10) |
| Region 3 | 4,091 (9) | 856 (11) | 157 (15) | 699 (10) | 3,235 (9) | 660 (10) | 2,575 (9) |
| Region 4 | 18,629 (43) | 4,213 (53) | 476 (46) | **3,737 (54)** | 14,416 (41) | 2,345 (37) | **12,071 (42)** |
| Region 5 | 3566 (8) | 547 (7) | 60 (6) | 487 (7) | 3,019 (9) | 382 (6) | 2637 (9) |
| Region 6 | 2,744 (6) | 420 (5) | 84 (8) | 336 (5) | 2,324 (7) | 606 (9) | 1,718 (6) |
| Region 7 | 3,458 (8) | 288 (4) | 52 (5) | **236 (3)** | 3,170 (9) | 665 (10) | **2,505 (9)** |
| Region 8 | 1,044 (2) | 105 (1) | 10 (1) | 95 (1) | 939 (3) | 148 (2) | 791 (3) |
| Region 9 | 2,070 (5) | 464 (6) | 48 (5) | 416 (6) | 1,606 (5) | 219 (3) | 1,387 (5) |
| Region 10 | 427 (1) | 38 (0) | 5 (0) | 33 (0) | 389 (1) | 80 (1) | 309 (1) |
| **Week of vaccination date** |  |  |  |  |  |  |  |
| Week of vaccination date, mean (SD) | 11.4 (4.4) | 10.8 (4.0) | 10.3 (3.2) | **10.9 (4.1)** | 11.5 (4.5) | 10.7 (3.8) | **11.7 (4.6)** |
| Week of vaccination date, median (IQR) | 11 (5) | 11 (5) | 10 (4) | 11 (5) | 11 (5) | 10 (5) | 11 (5) |
| **Test type, n (%)** |  |  |  |  |  |  |  |
| Antigen | 27,878 (65) | 4,981 (62) | 618 (60) | 4,363 (63) | 22,897 (65) | 4,295 (67) | 18,602 (65) |
| Molecular | 15,208 (35) | 3,014 (38) | 415 (40) | 2,599 (37) | 12,194 (35) | 2,095 (33) | 10,099 (35) |
| **Payer on influenza test date, n (%)** |  |  |  |  |  |  |  |
| Commercial | 19,119 (44) | 4,449 (56**)** | 617 (60) | **3,832 (55)** | 14,670 (42**)** | 2,728 (43) | **11,942 (42)** |
| Medicaid | 7,254 (17) | 891 (11) | 96 (9) | **795 (11)** | 6,363 (18) | 1,212 (19) | **5,151 (18)** |
| Medicare Advantage | 1,197 (3) | 328 (4) | 30 (3) | 298 (4) | 869 (2) | 86 (1) | 783 (3) |
| Unknown/other | 15,516 (36) | 2,327 (29) | 290 (28) | 2,037 (29) | 13,189 (38) | 2,364 (37) | 10,825 (38) |
| **COVID-19 vaccination during the 6 months prior to the test index date, n (%)** | 5,219 (12) | 1,400 (18) | 191 (18) | **1,209 (17)** | 3,819 (11) | 704 (11) | **3,115 (11)** |
| **Charlson Comorbidity Index** |  |  |  |  |  |  |  |
| Mean (SD) | 0.5 (1.1) | 0.6 (1.3) | 0.5 (1.0) | **0.6 (1.3)** | 0.4 (1.1) | 0.3 (0.8) | **0.5 (1.1)** |
| Median (IQR) | 0 (1) | 0 (1) | 0 (1) | 0 (1) | 0 (1) | 0 (0) | 0 (1) |
| **High-risk conditions, n (%)** |  |  |  |  |  |  |  |
| Asthma | 6,220 (14) | 1,117 (14) | 151 (15) | 966 (14) | 5,103 (15) | 926 (14) | 4,177 (15) |
| Neurologic and neurodevelopmental conditions | 2,993 (7) | 264 (3) | 39 (4) | **225 (3)** | 2,729 (8) | 564 (9) | **2,165 (8)** |
| Blood disorders | 5,142 (12) | 1,163 (15) | 113 (11) | 1,050 (15) | 3,979 (11) | 548 (9) | 3,431 (12) |
| Chronic lung disease | 1,493 (3) | 347 (4) | 32 (3) | 315 (5) | 1,146 (3) | 138 (2) | 1,008 (4) |
| Endocrine disorders | 7,958 (18) | 2,108 (26) | 194 (19) | **1,914 (27)** | 5,850 (17) | 731 (11) | **5,119 (18)** |
| Heart disease and related conditions | 10,785 (25) | 2,806 (35) | 298 (29) | **2,508 (36)** | 7,979 (23) | 1,027 (16) | **6,952 (24)** |
| Kidney diseases | 1,016 (2) | 255 (3) | 24 (2) | 231 (3) | 761 (2) | 82 (1) | 679 (2) |
| Liver disorders | 1,614 (4) | 434 (5) | 42 (4) | 392 (6) | 1,180 (3) | 125 (2) | 1,055 (4) |
| Metabolic disorders | 10,994 (26) | 2,978 (37) | 326 (32) | **2,652 (38)** | 8,016 (23) | 949 (15) | **7,067 (25)** |
| Obesity, BMI ≥40 kg/m^2^ | 3,589 (8) | 851 (11) | 91 (9) | 760 (11) | 2,738 (8) | 345 (5) | 2,393 (8) |
| Weakened immune system | 2,402 (6) | 612 (8) | 71 (7) | 541 (8) | 1,790 (5) | 232 (4) | 1,558 (5) |
| Stroke | 545 (1) | 144 (2) | 13 (1) | 131 (2) | 401 (1) | 43 (1) | 358 (1) |
| ≥1 High-risk condition | 23,472 (54) | 5,072 (63) | 567 (55) | **4,055 (65)** | 18,400 (52) | 2,872 (45) | **15,528 (54)** |
| **Baseline healthcare resource utilization** |  |  |  |  |  |  |  |
| No. of outpatient visits, mean (SD) | 6.5 (6.3) | 7.0 (6.9) | 6.2 (5.9) | 7.1 (7.0) | 6.4 (6.1) | 5.9 (5.6) | 6.5 (6.2) |
| No. of outpatient visits, median (IQR) | 5 (7) | 5 (7) | 5 (6) | 5 (8) | 5 (7) | 5 (6) | 5 (7) |
| No. of inpatient admissions, n (%) |  |  |  |  |  |  |  |
| 0 | 40,456 (94) | 7438 (93) | 993 (96) | 6,445 (93) | 33,018 (94) | 6,139 (96) | 26,879 (94) |
| 1 | 1,972 (5) | 392 (5) | 34 (3) | 358 (5) | 1,580 (5) | 201 (3) | 1,379 (5) |
| ≥2 | 658 (2) | 165 (2) | 6 (1) | 159 (2) | 493 (1) | 50 (1) | 443 (2) |
| No. of ED visits, n (%) |  |  |  |  |  |  |  |
| 0 | 33,971 (79) | 6,474 (81) | 860 (83) | 5,614 (81) | 27,497 (78) | 5,190 (81) | 22,307 (78) |
| 1 | 5,642 (13) | 914 (11) | 113 (11) | 801 (12) | 4,728 (13) | 794 (12) | 3,934 (14) |
| ≥2 | 3,473 (8) | 607 (8) | 60 (6) | 547 (8) | 2,866 (8) | 406 (6) | 2,460 (9) |

BMI, body mass index; ED, emergency department; HHS, U.S. Department of Health and Human Services; IQR, interquartile range; QIVc, cell-based quadrivalent influenza vaccine; QIVe, egg-based quadrivalent influenza vaccine; SD, standard deviation; SMD, standardized mean difference.

^a^Control group baseline characteristics with an unweighted SMD >0.1 between the QIVc and QIVe groups are highlighted in bold.

Figure S2. Covariate balance of controls in the matched on week population before and after weighting

BMI, body mass index; CCI, Charlson comorbidity index; ER, emergency room/department; HHS, U.S. Department of Health and Human Services; IP, inpatient.


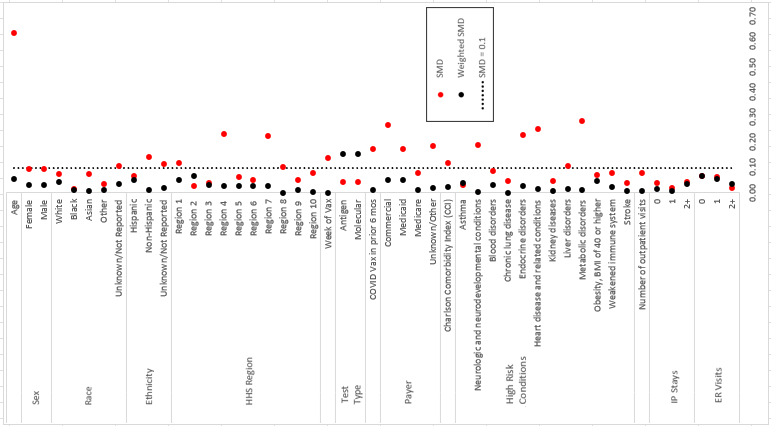


Table S8. Demographic and clinical characteristics of the peak season study population

| **Variable** | **Total**  **(n=17,500)** | **QIVc** | | **QIVe** | |
| --- | --- | --- | --- | --- | --- |
|  |  | **Case**  **(n=641)** | **Control**  **(n=2,549)** | **Case**  **(n=4,078)** | **Control**  **(n=10,232)** |
| **Percent of original population** | **41%** | **62%** | **37%** | **64%** | **36%** |
| **Week of vaccination date** |  |  |  |  |  |
| Week of vaccination date, mean (SD) | 10.3 (2.9) | 10.1 (2.9) | 10.4 (2.9) | 10.3 (2.9) | 10.1 (2.9) |
| Week of vaccination date, median (IQR) | 10 (4) | 10 (4) | 10 (4) | 10 (4) | 10 (4) |
| **Test type, n (%)** |  |  |  |  |  |
| Antigen | 11,194 (64) | 1,967 (62) | 9,227 (64) | 11,194 (64) | 1,967 (62) |
| Molecular | 6,306 (36) | 1,223 (38) | 5,083 (36) | 6,306 (36) | 1,223 (38) |
| **COVID-19 vaccination during the 6 months prior to the test index date, n (%)** | 2,218 (13) | 530 (17) | 1,688 (12) | 2,218 (13) | 530 (17) |
| **Age at index date, years, mean (SD)** | 25.8 (21.5) | 30.1 (20.9) | 38.1 (19.5) | 18.7 (18.8) | 25.2 (21.7) |
| Age at index date, years, median (IQR) | 16 (40) | 26 (41) | 42 (36) | 10 (22) | 16 (40) |
| **Age group at index date, n (%)** |  |  |  |  |  |
| 0-4 years | 2,978 (17) | 23 (4) | 108 (4) | 737 (18) | 2,110 (21) |
| 5-17 years | 6,214 (36) | 271 (42) | 468 (18) | 2,158 (53) | 3,317 (32) |
| 18-49 years | 4,406 (25) | 170 (27) | 1,027 (40) | 650 (16) | 2,559 (25) |
| 50-64 years | 3,902 (22) | 177 (28) | 946 (37) | 533 (13) | 2,246 (22) |
| **Sex, n (%)** |  |  |  |  |  |
| Female | 10,272 (59) | 361 (56) | 1,628 (64) | 2,203 (54) | 6,080 (59) |
| Male | 7,228 (41) | 280 (44) | 921 (36) | 1,875 (46) | 4,152 (41) |
| **Race, n (%)** |  |  |  |  |  |
| White | 12,610 (72) | 495 (77) | 1,907 (75) | 2,907 (71) | 7,301 (71) |
| Black | 1,169 (7) | 32 (5) | 172 (7) | 271 (7) | 694 (7) |
| Asian | 488 (3) | 26 (4) | 96 (4) | 105 (3) | 261 (3) |
| Other | 1,000 (6) | 33 (5) | 117 (5) | 248 (6) | 602 (6) |
| Unknown/not reported | 2,233 (13) | 55 (9) | 257 (10) | 547 (13) | 1,374 (13) |
| **Ethnicity, n (%)** |  |  |  |  |  |
| Hispanic | 1,364 (8) | 31 (5) | 158 (6) | 396 (10) | 779 (8) |
| Non-Hispanic | 12,474 (71) | 486 (76) | 1,952 (77) | 2,835 (70) | 7,201 (70) |
| Unknown/not reported | 3,662 (21) | 124 (19) | 439 (17) | 847 (21) | 2,252 (22) |
| **HHS region, n (%)** |  |  |  |  |  |
| Region 1: Connecticut, Maine, Massachusetts, New Hampshire, Rhode Island, Vermont | 1,266 (7) | 41 (6) | 95 (4) | 455 (11) | 675 (7) |
| Region 2: New Jersey, New York, Puerto Rico, the Virgin Islands | 1,694 (10) | 58 (9) | 222 (9) | 453 (11) | 961 (9) |
| Region 3: Delaware, District of Columbia, Maryland, Pennsylvania Virginia, West Virginia | 1,645 (9) | 101 (16) | 282 (11) | 418 (10) | 844 (8) |
| Region 4: Alabama, Florida, Georgia, Kentucky, Mississippi, North Carolina, South Carolina, Tennessee | 7,195 (41) | 270 (42) | 1,342 (53) | 1,353 (33) | 4,230 (41) |
| Region 5: Illinois, Indiana, Michigan, Minnesota, Ohio, Wisconsin | 1,322 (8) | 36 (6) | 162 (6) | 252 (6) | 872 (9) |
| Region 6: Arkansas, Louisiana, New Mexico, Oklahoma, Texas | 1,168 (7) | 49 (8) | 143 (6) | 327 (8) | 649 (6) |
| Region 7: Iowa, Kansas, Missouri, Nebraska | 1,721 (10) | 43 (7) | 110 (4) | 496 (12) | 1,072 (10) |
| Region 8: Colorado, Montana, North Dakota, South Dakota, Utah, Wyoming | 463 (3) | 9 (1) | 43 (2) | 98 (2) | 313 (3) |
| Region 9: Arizona, California, Hawaii, Nevada, American Samoa, Commonwealth of the Norther Mariana Islands, Federated States of Micronesia, Guam, Marshall Islands, Republic of Palau | 808 (5) | 30 (5) | 138 (5) | 161 (4) | 479 (5) |
| Region 10: Alaska, Idaho, Oregon, Washington | 218 (1) | 4 (1) | 12 (0) | 65 (2) | 137 (1) |
| **Payer on influenza test date, n (%)** |  |  |  |  |  |
| Commercial | 8,168 (47) | 1,832 (57) | 6,336 (44) | 8,168 (47) | 1,832 (57) |
| Medicaid | 2,770 (16) | 334 (10) | 2,436 (17) | 2,770 (16) | 334 (10) |
| Medicare Advantage | 428 (2) | 118 (4) | 310 (2) | 428 (2) | 118 (4) |
| Unknown/other | 6,134 (35) | 906 (28) | 5,228 (37) | 6,134 (35) | 906 (28) |
| **Charlson Comorbidity Index** |  |  |  |  |  |
| Mean (SD) | 0.4 (1.1) | 0.4 (0.9) | 0.6 (1.3) | 0.3 (0.8) | 0.5 (1.1) |
| Median (IQR) | 0 (1) | 0 (1) | 0 (1) | 0 (0) | 0 (1) |
| **High risk conditions, n (%)** |  |  |  |  |  |
| Asthma | 2,614 (15) | 104 (16) | 349 (14) | 607 (15) | 1,554 (15) |
| Neurologic and neurodevelopmental conditions | 1,276 (7) | 28 (4) | 94 (4) | 349 (9) | 805 (8) |
| Blood disorders | 1,897 (11) | 62 (10) | 359 (14) | 319 (8) | 1,157 (11) |
| Chronic lung disease | 545 (3) | 18 (3) | 101 (4) | 82 (2) | 344 (3) |
| Endocrine disorders | 3,033 (17) | 109 (17) | 676 (27) | 442 (11) | 1,806 (18) |
| Heart disease and related conditions | 4,084 (23) | 171 (27) | 902 (35) | 622 (15) | 2,389 (23) |
| Kidney diseases | 351 (2) | 13 (2) | 74 (3) | 46 (1) | 218 (2) |
| Liver disorders | 586 (3) | 20 (3) | 132 (5) | 70 (2) | 364 (4) |
| Metabolic disorders | 4125 (24) | 181 (28) | 935 (37) | 577 (14) | 2432 (24) |
| Obesity, BMI ≥40 kg/m^2^ | 1349 (8) | 54 (8) | 275 (11) | 203 (5) | 817 (8) |
| Weakened immune system | 917 (5) | 41 (6) | 179 (7) | 147 (4) | 550 (5) |
| Stroke | 189 (1) | 8 (1) | 46 (2) | 20 (0) | 115 (1) |
| **Baseline healthcare resource utilization** |  |  |  |  |  |
| No. of outpatient visits, mean (SD) | 6.5 (6.1) | 6.2 (5.8) | 7.0 (6.6) | 5.8 (5.4) | 6.7 (6.2) |
| No. of outpatient visits, median (IQR) | 5 (7) | 5 (6) | 5 (7) | 4 (6) | 5 (7) |
| No. of inpatient admissions, n (%) | ( ) | ( ) | ( ) | ( ) | ( ) |
| 0 | 16,582 (95) | 617 (96) | 2,382 (93) | 3,934 (96) | 9,649 (94) |
| 1 | 697 (4) | 21 (3) | 126 (5) | 111 (3) | 439 (4) |
| ≥2 | 221 (1) | 3 (0) | 41 (2) | 33 (1) | 144 (1) |
| No. of ED visits, n (%) |  |  |  |  |  |
| 0 | 13,954 (80) | 527 (82) | 2,061 (81) | 3,336 (82) | 8,030 (78) |
| 1 | 2,241 (13) | 77 (12) | 297 (12) | 491 (12) | 1,376 (13) |
| ≥2 | 1,305 (7) | 37 (6) | 191 (7) | 251 (6) | 826 (8) |

BMI, body mass index; ED, emergency department; HHS, U.S. Department of Health and Human Services; IQR, interquartile range; QIVc, cell-based quadrivalent influenza vaccine; QIVe, egg-based quadrivalent influenza vaccine; SD, standard deviation.

Figure S3. Covariate balance of controls in the peak period population before and after weighting

BMI, body mass index; CCI, Charlson comorbidity index; ER, emergency room/department; HHS, U.S. Department of Health and Human Services; IP, inpatient.


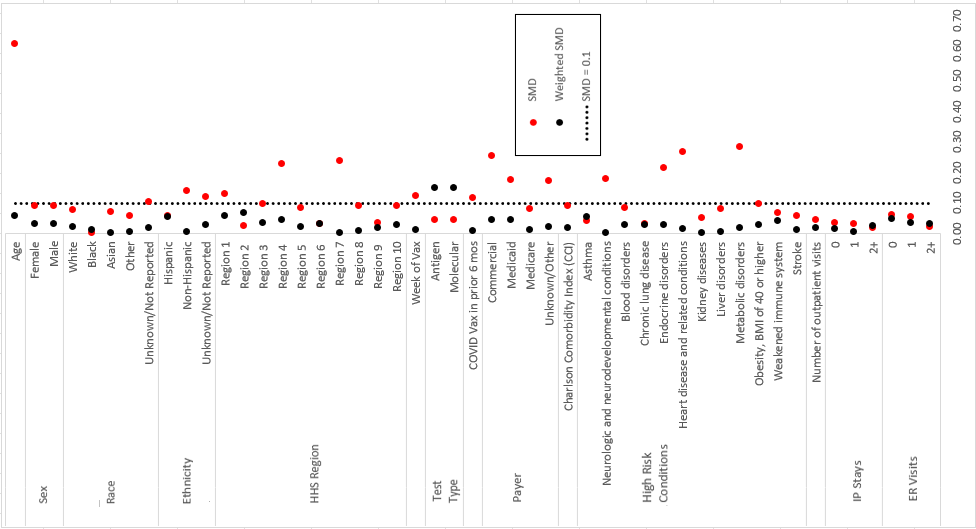


Table S9. Additional outcomes prevented by use of QIVc vs. QIVe, by age group

| **Outcome** | **0–4 Years** | **5–17 Years** | **18–49 Years** | **50–64 Years** | **Total** |
| --- | --- | --- | --- | --- | --- |
| **Averted with QIVc** | | | | | |
| Symptomatic cases | 1,102,990 | 2,350,182 | 1,452,626 | 1,344,069 | 6,249,867 |
| Outpatient visits | 739,003 | 1,222,095 | 537,472 | 577,950 | 3,076,520 |
| Hospitalizations^a^ | 7,689 | 6,444 | 8,154 | 14,253 | 36,540 |
| ICU visits | 1,169 | 979 | 1,239 | 2,167 | 5,554 |
| Deaths | 75 | 65 | 131 | 968 | 1,239 |
| **Averted with QIVe** | | | | | |
| Symptomatic cases | 986,810 | 2,114,363 | 1,310,179 | 1,202,306 | 5,613,658 |
| Outpatient visits | 661,163 | 1,099,468 | 484,766 | 516,992 | 2,762,389 |
| Hospitalizations^a^ | 6,880 | 5,797 | 7,354 | 12,750 | 32,781 |
| ICU visits | 1,046 | 881 | 1,118 | 1,938 | 4,983 |
| Deaths | 67 | 58 | 118 | 866 | 1,109 |
| **Incremental cases averted** | | | | | |
| Symptomatic cases | 116,180 | 235,820 | 142,447 | 141,763 | 636,210 |
| Outpatient visits | 77,840 | 122,626 | 52,705 | 60,958 | 314,129 |
| Hospitalizations^a^ | 810 | 647 | 800 | 1,503 | 3,760 |
| ICU visits | 123 | 98 | 122 | 229 | 572 |
| Deaths | 8 | 6 | 13 | 102 | 129 |

ICU, intensive care unit; QIVc, cell-based quadrivalent influenza vaccine; QIVe, egg-based cell-based quadrivalent influenza vaccine.

^a^Hospitalizations include both ICU and non-ICU hospital stays.

Figure S4. Deterministic sensitivity analysis of uncertainty around the base-case results of the analysis of symptomatic cases averted

The deterministic sensitivity analysis (DSA) evaluated the impact that individual model parameters had on the results by evaluating the change in the number of outcomes prevented from base case analysis when using the upper and lower limits of the 95% confidence intervals of the estimates instead of the point estimate. (A) Symptomatic cases. (B) Outpatient visits. (C) Hospitalizations. (D) Deaths.


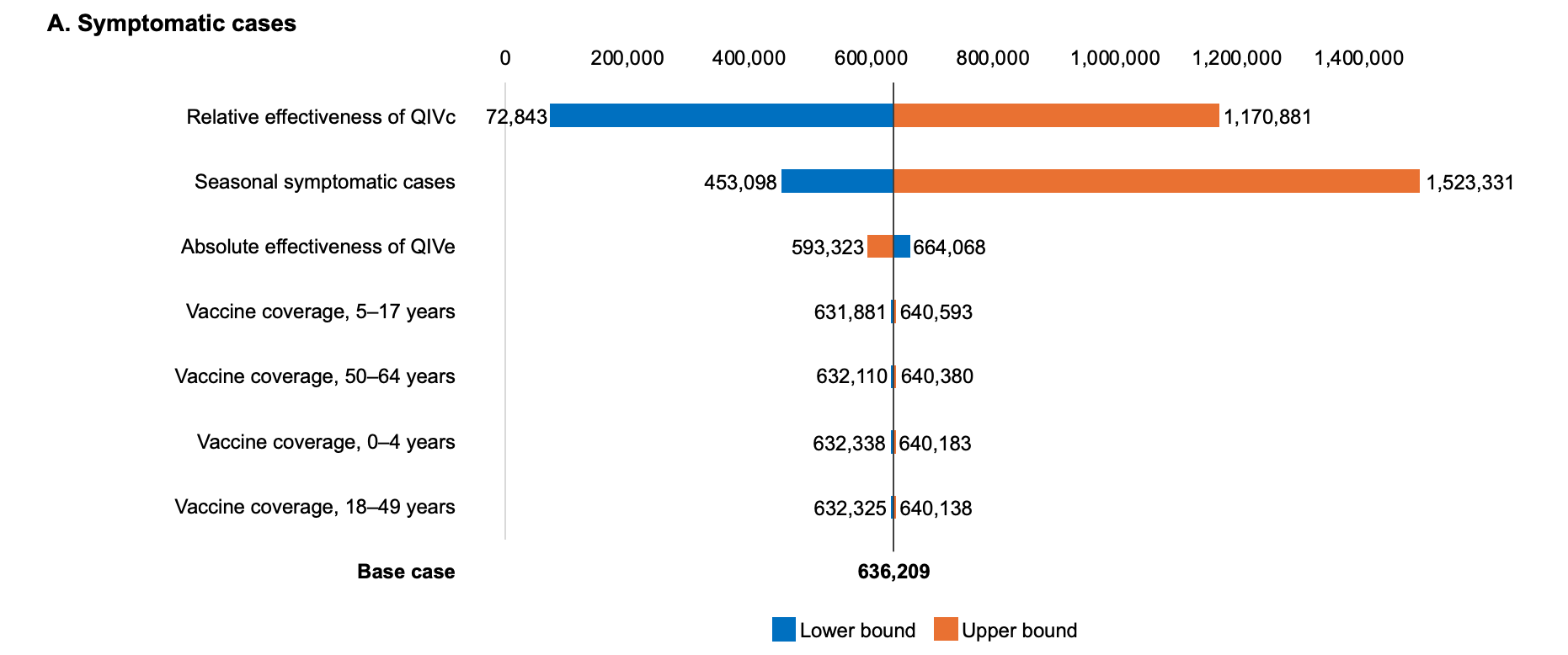


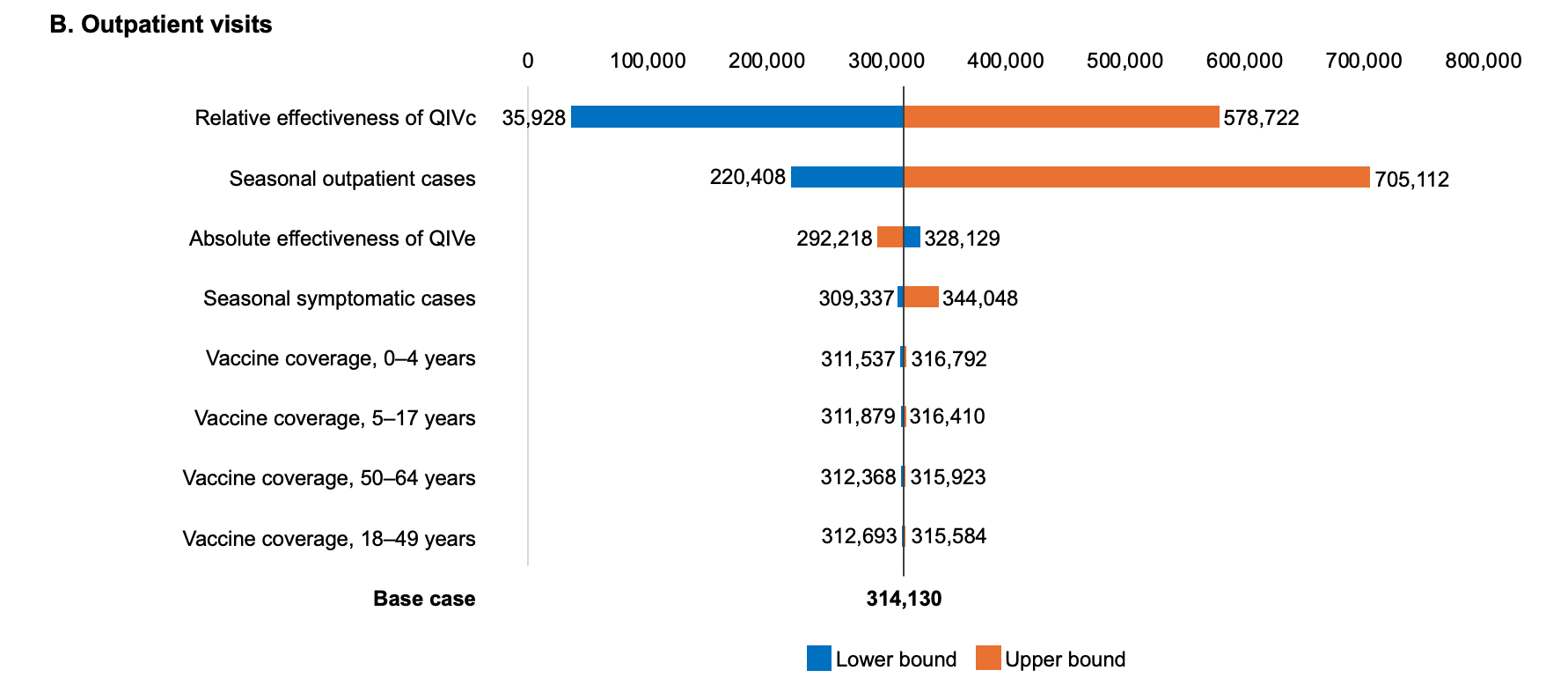


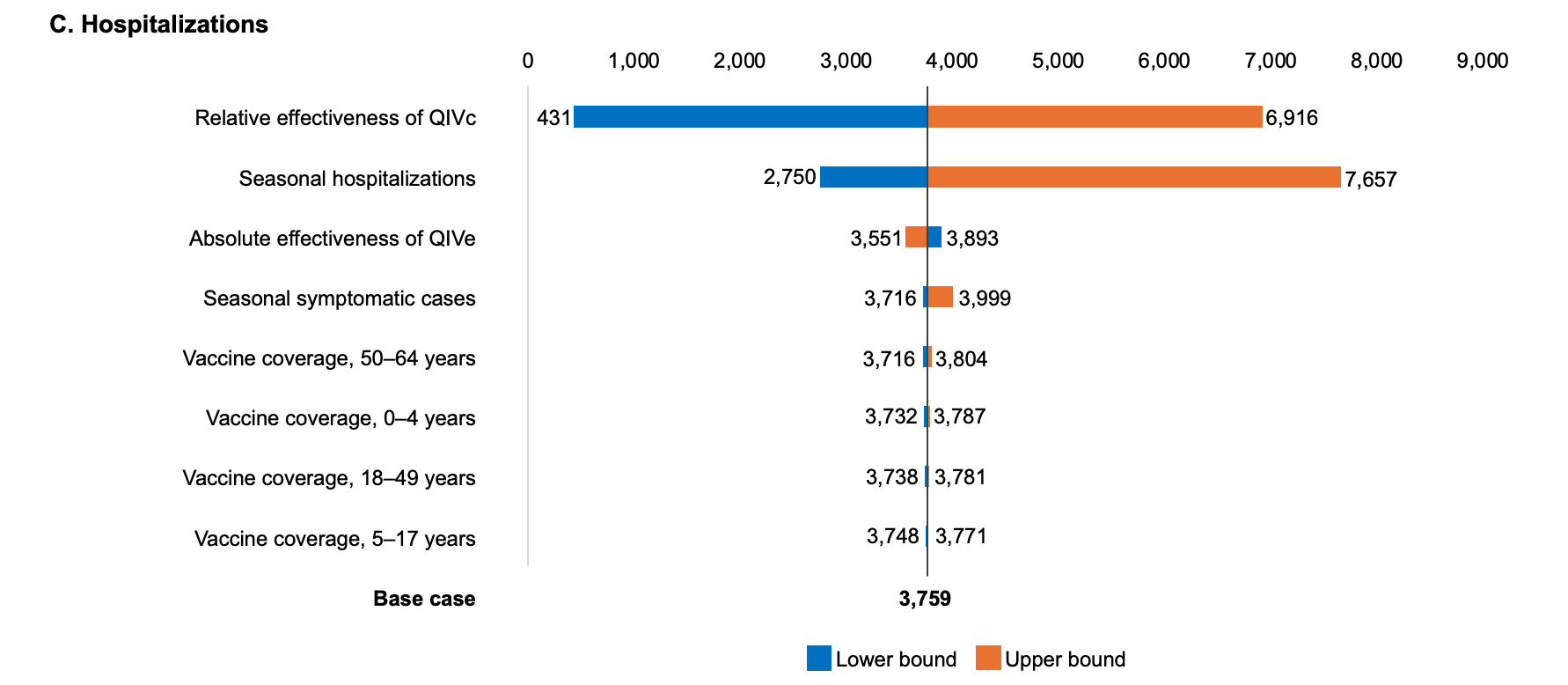


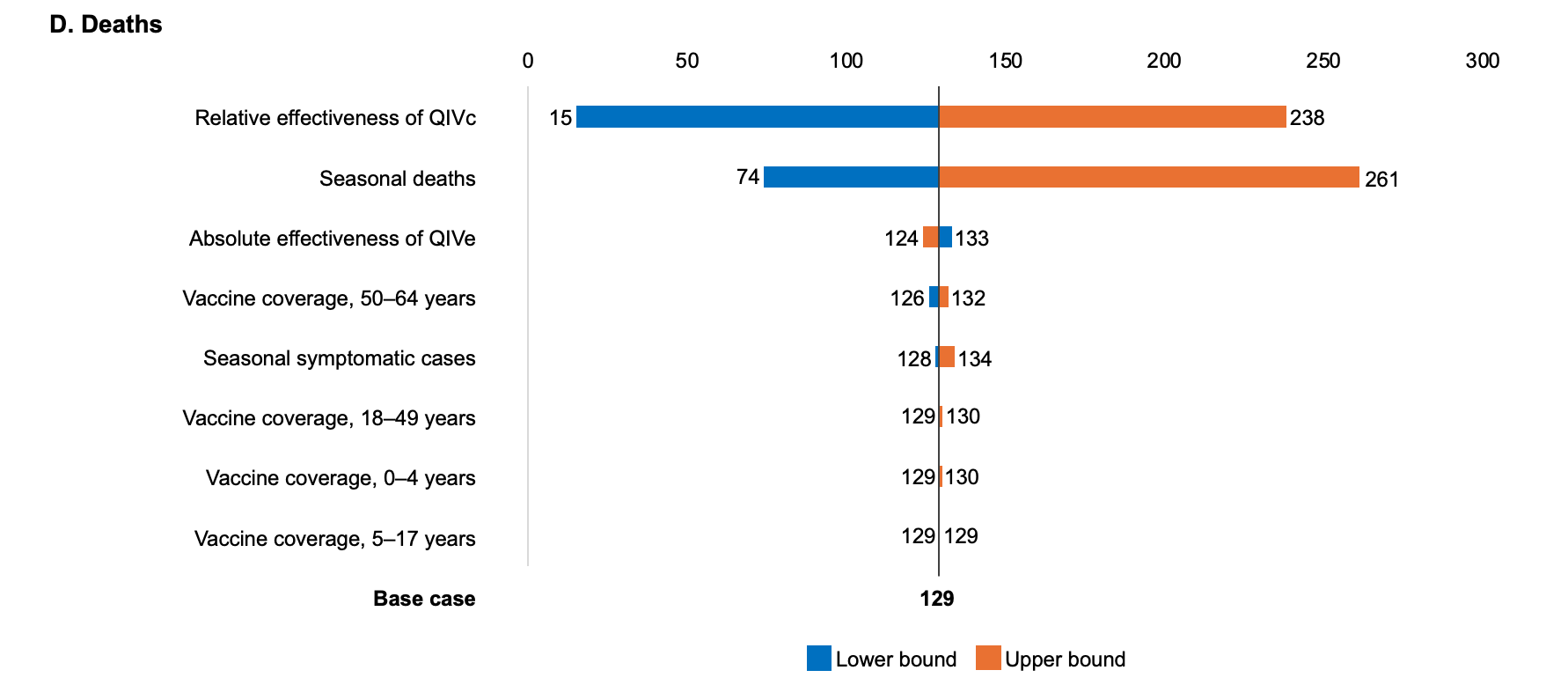


Figure S5. Probabilistic sensitivity analysis

The probabilistic sensitivity analysis (PSA) evaluated the distribution of expected results after repeatedly resampling the input parameters over an assumed distribution. The center blue line represents the incremental mean, and the lower and upper bounds of the box represent the first and third quartiles of the incremental results, respectively. Dots represent individual observations, and whiskers represent 1.5 times the inter-quartile range. (A) Symptomatic cases. (B) Outpatient visits. (C) Hospitalizations. (D) Deaths.

| 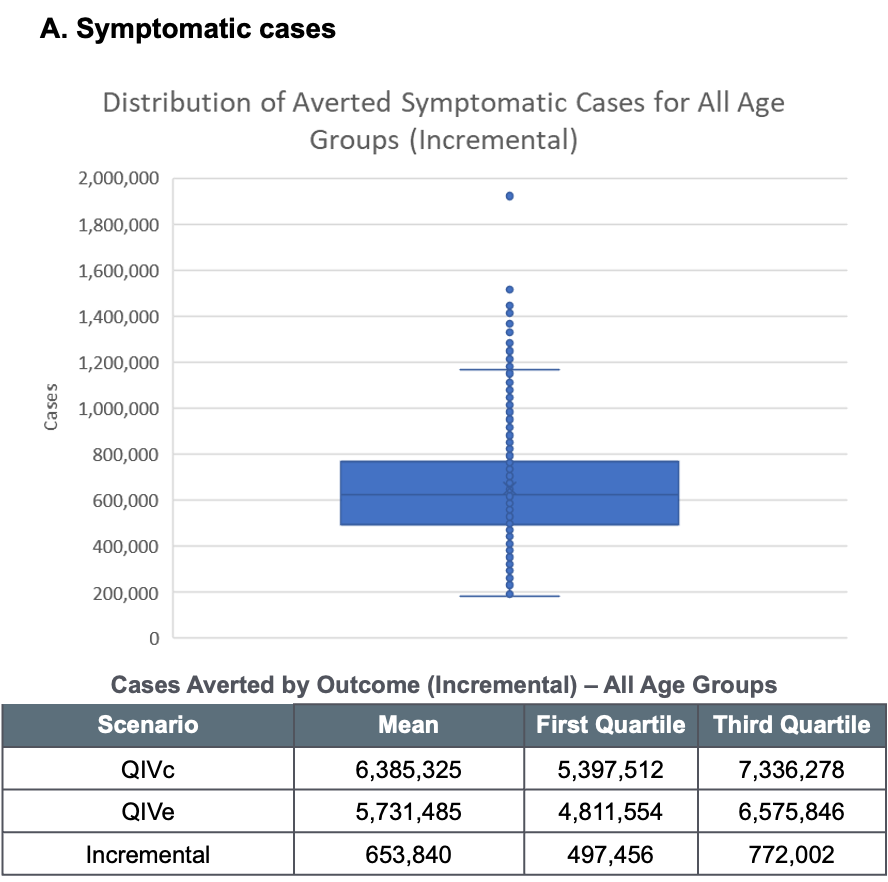 | 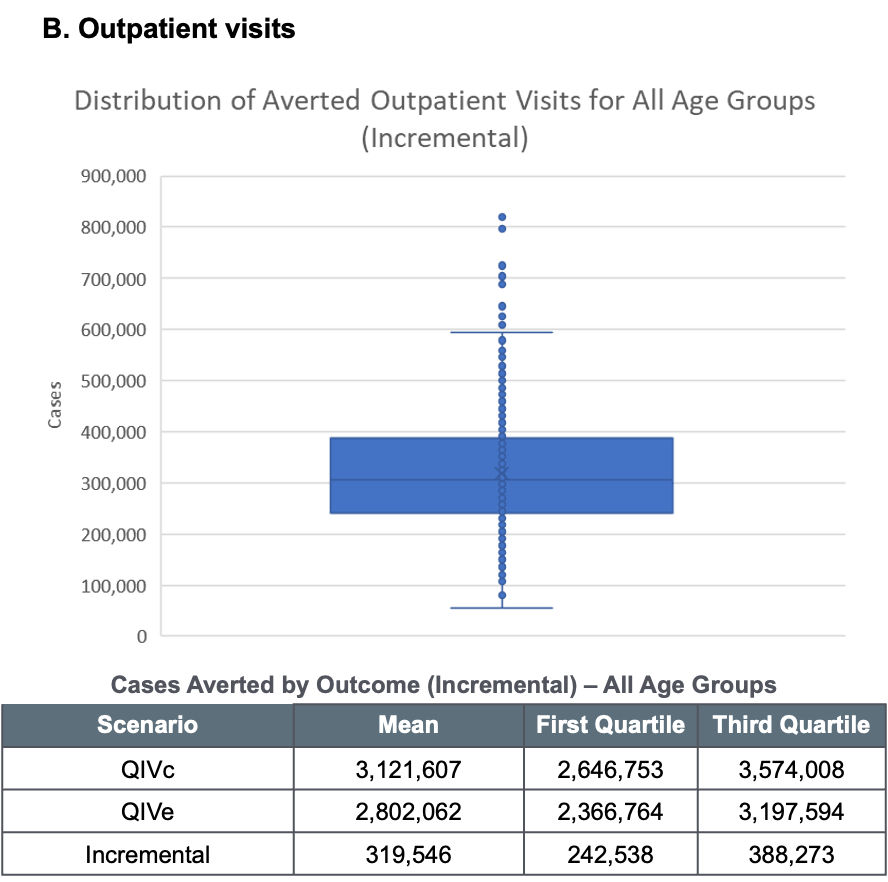 |
| --- | --- |
| 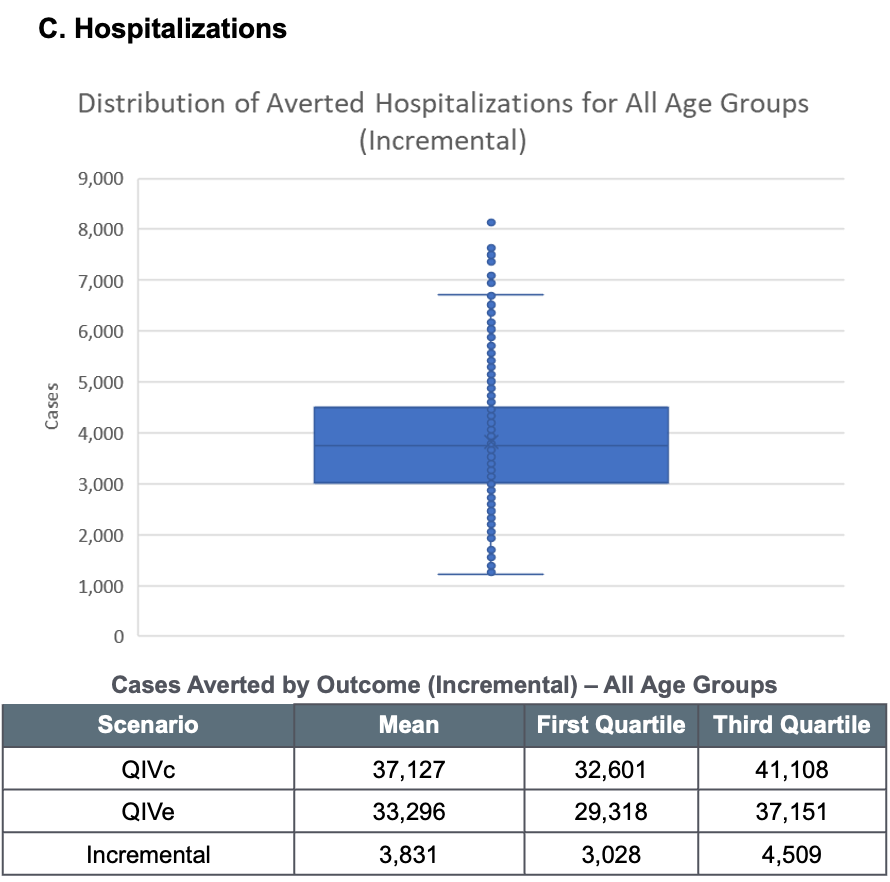 | 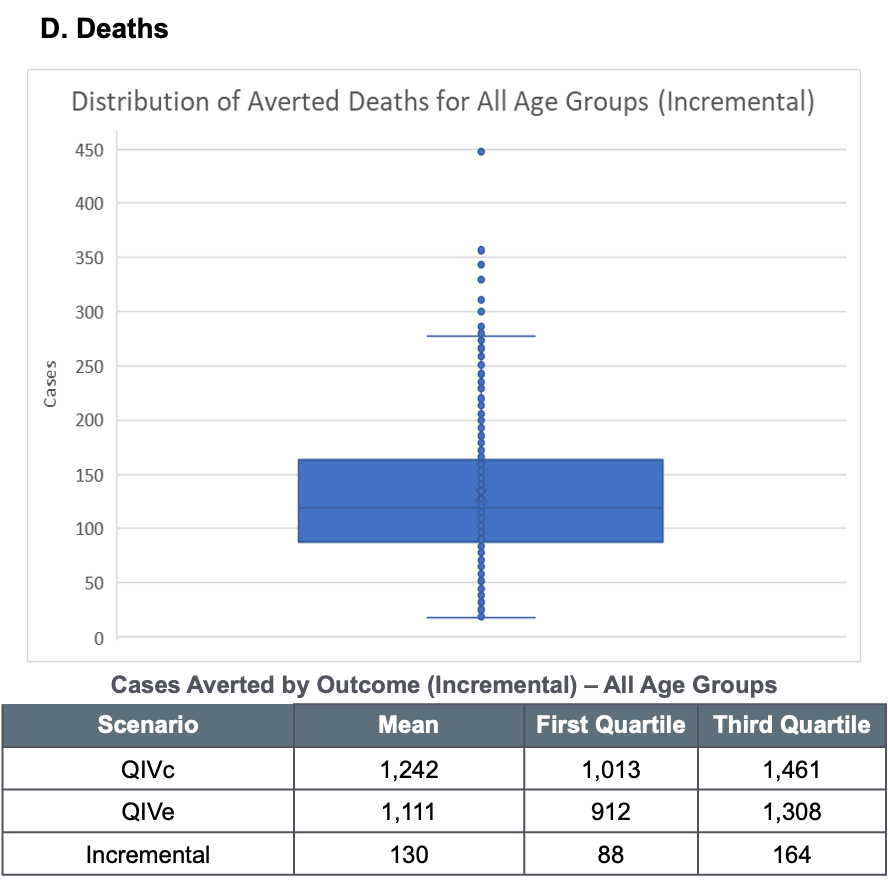 |

Table S10. Amino acid substitutions in the HA protein of cell-based and egg-based vaccine viruses, NH 2022–2023 season

| **Strain** | **Cell-based vaccine** | | **Egg-based vaccine** | |
| --- | --- | --- | --- | --- |
|  | **WHO recommended strain and CVVs^a^** | **Amino acid changes in HA protein^b^** | **WHO recommended strain and CVVs^a^** | **Amino acid changes in HA protein**^c, d^ |
| A/H1N1 | ***A/Wisconsin/588/2019 (H1N1)pdm09 -like virus***  A/Delaware/55/2019 CVR-45 | No amino acid changes | ***A/Victoria/2570/2019 (H1N1)pdm09-like virus***  A/Indiana/02/2020  A/Victoria/2570/2019  A/Victoria/3/2020  A/Victoria/1/2020 | V19I, Q223R  A195E, Q223R  K112E, Q223R  K112E, Q223R |
| A/H3N2 | ***A/Darwin/6/2021 (H3N2)-like virus***  A/Darwin/11/2021 | No amino acid changes | ***A/Darwin/9/2021 (H3N2)-like virus***  A/Darwin/9/2021  A/Darwin/6/2021  A/Michigan/173/2020  A/Netherlands/00007/2021  A/Alaska/01/2021 | S46P, D186N, D225G  D186N, D225G  D186N, D225G  D186N, D225G  S96C, D186N, D225G |
| B/Victoria | ***B/Austria/1359417/2021 (B/Victoria lineage)-like virus***  B/Singapore/WUH4618/2021 | No amino acid changes | ***B/Austria/1359417/2021 (B/Victoria lineage)-like virus***  B/Austria/1359417/2021  B/Michigan/01/2021  B/Singapore/WUH4618/2021  B/GuangdongZhenjiang/1516/2021  B/Zhejiang-Nanhu/1854/2021  B/ZhejiangXiacheng/11085/2021  B/Shaanxi-Baota/1278/2022 | G141R  G141R  No amino acid changes  D194Y, T232I  G141R  G141R  G141R |
| B/Yamagata | ***B/Phuket/3073/2013 B/Yamagata lineage)-like virus***  B/Singapore/INFTT-16-0610/2016 | No amino acid changes | ***B/Phuket/3073/2013 (B/Yamagata lineage)-like virus***  B/Phuket/3073/2013  B/Brisbane/9/2014  B/Utah/09/2014  B/California/12/2015  B/Arizona/10/2015  B/Hong Kong/432/2013  B/Hong Kong/3417/2014 | N197D, G238E  N197D  V91I, N197E, N197I, N197K  G142E, T199I K163E, N197D, N197S, S457G N197K, T199N, T260K T199N, T260K |

CVV, candidate vaccine virus; HA, hemagglutinin; NH, Northern Hemisphere; WHO, World Health Organization.

^a^All viruses that have been through manufacturing as listed by the WHO^23^ for the 2022–2023 season are included.

^b^Analyses compared working virus seed to original clinical sample.^24^

^c^Amino acid change in at least one wild type or reassortant vaccine virus identified from a detailed sequence analysis performed by CSL Seqirus.^25^

^d^Amino acid substitutions in the HA protein of the egg-based vaccines (egg-adapted mutations) are highlighted in colour based on their location: For A/H1N1: yellow: receptor binding site; pink: antigenic site Sb; For A/H3N2: yellow: receptor binding site; red: antigenic site B; dark yellow: antigenic site C; green: antigenic site D; For B viruses: dark red: antigenic site (150 loop); dark blue: antigenic site (160 loop); turquoise: antigenic site (190 helix).

**References**

1. Glasheen WP, Cordier T, Gumpina R, Haugh G, Davis J, Renda A. Charlson Comorbidity Index: ICD-9 Update and ICD-10 Translation. *American health & drug benefits.* 2019;12(4):188-197.

2. Grohskopf LA, Blanton LH, Ferdinands JM, et al. Prevention and Control of Seasonal Influenza with Vaccines: Recommendations of the Advisory Committee on Immunization Practices - United States, 2022-23 Influenza Season. *MMWR Recomm Rep.* 2022;71(1):1-28.

3. Centers for Disease Control and Prevention. People at increased risk for flu complications. 2024; <https://www.cdc.gov/flu/highrisk/>. Accessed 22 November 2024.

4. Austin PC. An Introduction to Propensity Score Methods for Reducing the Effects of Confounding in Observational Studies. *Multivariate Behav Res.* 2011;46(3):399-424.

5. Rothman KJ, Greenland S. Basic methods for sensitivity analysis and external adjustment. In: *Modern Epidemiology.* 2nd ed. Philadelphia: Lippincott Williams & Wilkins; 1998:343-347.

6. Chua H, Feng S, Lewnard JA, et al. The Use of Test-negative Controls to Monitor Vaccine Effectiveness: A Systematic Review of Methodology. *Epidemiology.* 2020;31(1):43-64.

7. Vandenbroucke JP, Pearce N. Test-Negative Designs: Differences and Commonalities with Other Case-Control Studies with "Other Patient" Controls. *Epidemiology.* 2019;30(6):838-844.

8. Rutherford MJ, Crowther MJ, Lambert PC. The use of restricted cubic splines to approximate complex hazard functions in the analysis of time-to-event data: a simulation study. *Journal of Statistical Computation and Simulation.* 2015;85(4):777-793.

9. Kuo CL, Duan Y, Grady J. Unconditional or Conditional Logistic Regression Model for Age-Matched Case-Control Data? *Frontiers in public health.* 2018;6:57.

10. Greifer N, Stuart EA. Matching Methods for Confounder Adjustment: An Addition to the Epidemiologist's Toolbox. *Epidemiol Rev.* 2022;43(1):118-129.

11. Rassen JA, Shelat AA, Myers J, Glynn RJ, Rothman KJ, Schneeweiss S. One-to-many propensity score matching in cohort studies. *Pharmacoepidemiology and drug safety.* 2012;21 Suppl 2:69-80.

12. Rubin DB, Thomas N. Matching using estimated propensity scores: relating theory to practice. *Biometrics.* 1996;52(1):249-264.

13. Trombetta VK, Chan YL, Bankowski MJ. Are Rapid Influenza Antigen Tests Still Clinically Useful in Today's Molecular Diagnostics World? *Hawaii J Med Public Health.* 2018;77(9):226-230.

14. Parikh R, Mathai A, Parikh S, Chandra Sekhar G, Thomas R. Understanding and using sensitivity, specificity and predictive values. *Indian J Ophthalmol.* 2008;56(1):45-50.

15. Vega T, Lozano JE, Meerhoff T, et al. Influenza surveillance in Europe: establishing epidemic thresholds by the moving epidemic method. *Influenza Other Respir Viruses.* 2013;7(4):546-558.

16. Centers for Disease Control and Prevention. FluView interactive: national, regional, and state level outpatient illness and viral surveillance. <https://gis.cdc.gov/grasp/fluview/fluportaldashboard.html>. Accessed 7 November 2024.

17. Malosh RE, McGovern I, Monto AS. Influenza During the 2010-2020 Decade in the United States: Seasonal Outbreaks and Vaccine Interventions. *Clinical infectious diseases : an official publication of the Infectious Diseases Society of America.* 2023;76(3):540-549.

18. Boikos C, McGovern I, Ortiz JR, Puig-Barberà J, Versage E, Haag M. Relative Vaccine Effectiveness of Adjuvanted Trivalent Influenza Vaccine over Three Consecutive Influenza Seasons in the United States. *Vaccines (Basel).* 2022;10(9).

19. Centers for Disease Control and Prevention. Flu vaccine effectiveness (VE) data for 2022-2023. 2024; <https://www.cdc.gov/flu-vaccines-work/php/effectiveness-studies/2022-2023.html>. Accessed 23 June 2025.

20. Centers for Disease Control and Prevention. Flu vaccination coverage, United States, 2022–2023 influenza season. 2024; <https://www.cdc.gov/fluvaxview/coverage-by-season/2022-2023.html>. Accessed 22 November 2024.

21. Centers for Disease Control and Prevention. Preliminary Estimated Flu Disease Burden 2022–2023 Flu Season. 2023; <https://www.cdc.gov/flu-burden/php/data-vis/2022-2023.html>. Accessed 22 November 2024.

22. Centers for Disease Control and Prevention. Laboratory-confirmed influenza hospitalizations, 2022-2023. 2024; <https://gis.cdc.gov/grasp/fluview/FluHospChars.html>. Accessed 6 December 2024.

23. World Health Organization Global Influenza Programme. Candidate vaccine viruses and potency testing reagents. 2025; <https://www.who.int/teams/global-influenza-programme/vaccines/who-recommendations/candidate-vaccine-viruses>. Accessed 16 September 2025.

24. Rockman S, Laurie K, Ong C, et al. Cell-Based Manufacturing Technology Increases Antigenic Match of Influenza Vaccine and Results in Improved Effectiveness. *Vaccines (Basel).* 2022;11(1).

25. Ashraf M, Stein AN, Youhanna J, et al. The impact of egg adaptation and immune imprinting on influenza vaccine effectiveness. *Vaccine.* 2025;62:127393.

1. In the United States, the Centers for Disease Control and Prevention (CDC) employs a system known as the Influenza-Like Illness (ILI) Activity Level Indicator to assess flu activity. This system evaluates the proportion of outpatient visits for ILI relative to a baseline, which is established using historical data. Researchers then determine a threshold, defining x% above the baseline as ‘high’ intensity. This intensity level is recorded based on the calendar time during which it occurs. Finally, the positivity rates at the beginning and end of this period are reported. [↑](#footnote-ref-1)
